# Non-Peptide Opioids Differ in Effects on Mu-Opioid (MOP) and Serotonin 1A (5-HT_1A_) Receptors Heterodimerization and Cellular Effectors (Ca^2+^, ERK1/2 and p38) Activation

**DOI:** 10.3390/molecules27072350

**Published:** 2022-04-06

**Authors:** Vlad Radoi, Gerd Jakobsson, Vinko Palada, Andrej Nikosjkov, Henrik Druid, Lars Terenius, Eva Kosek, Vladana Vukojević

**Affiliations:** 1 Department of Clinical Neuroscience, Karolinska Institute, 171 76 Stockholm, Sweden; vlad.radoi@su.se (V.R.); andrej.nikosjkov@ki.se (A.N.); lars.terenius@ki.se (L.T.); eva.kosek@ki.se (E.K.); 2 Department of Forensic Genetics and Forensic Toxicology, National Board of Forensic Medicine, 587 58 Linköping, Sweden; gerd.jakobsson@liu.se (G.J.); henrik.druid@ki.se (H.D.); 3 Department of Physiology, SleepWell Research Program, Faculty of Medicine, University of Helsinki, 00290 Helsinki, Finland; vinko.palada@helsinki.fi; 4 Department of Oncology-Pathology, Karolinska Institute, 171 77 Stockholm, Sweden; 5 Department of Surgical Sciences, Uppsala University, 752 36 Uppsala, Sweden

**Keywords:** chronic pain, fluorescence cross-correlation spectroscopy (FCCS), G protein-coupled receptor (GPCR), opioid, serotonin

## Abstract

The importance of the dynamic interplay between the opioid and the serotonin neuromodulatory systems in chronic pain is well recognized. In this study, we investigated whether these two signalling pathways can be integrated at the single-cell level via direct interactions between the mu-opioid (MOP) and the serotonin 1A (5-HT_1A_) receptors. Using fluorescence cross-correlation spectroscopy (FCCS), a quantitative method with single-molecule sensitivity, we characterized in live cells MOP and 5-HT_1A_ interactions and the effects of prolonged (18 h) exposure to selected non-peptide opioids: morphine, codeine, oxycodone and fentanyl, on the extent of these interactions. The results indicate that in the plasma membrane, MOP and 5-HT_1A_ receptors form heterodimers that are characterized with an apparent dissociation constant $K_{d}^{\mathrm{app}}$ = (440 ± 70) nM). Prolonged exposure to all non-peptide opioids tested facilitated MOP and 5-HT_1A_ heterodimerization and stabilized the heterodimer complexes, albeit to a different extent: $K_{d,\;\mathrm{Fentanyl}}^{\mathrm{app}}$ = (80 ± 70) nM), $K_{d,\mathrm{Morphine}}^{\mathrm{app}}$ = (200 ± 70) nM, $K_{d,\;\mathrm{Codeine}}^{\mathrm{app}}$ = (100 ± 70) nM and $K_{d,\;\mathrm{Oxycodone}}^{\mathrm{app}}$ = (200 ± 70) nM. The non-peptide opioids differed also in the extent to which they affected the mitogen-activated protein kinases (MAPKs) p38 and the extracellular signal-regulated kinase (Erk1/2), with morphine, codeine and fentanyl activating both pathways, whereas oxycodone activated p38 but not ERK1/2. Acute stimulation with different non-peptide opioids differently affected the intracellular Ca^2+^ levels and signalling dynamics. Hypothetically, targeting MOP–5-HT_1A_ heterodimer formation could become a new strategy to counteract opioid induced hyperalgesia and help to preserve the analgesic effects of opioids in chronic pain.

## 1. Introduction

Chronic pain is a major health issue worldwide [1] that enacts considerable suffering [2,3]. Despite the limited effects of available drugs for the treatment of pain, chronic pain patients are often treated with opioids, which have a controversial role in chronic pain management. In fact, patient follow-ups and population studies reveal the low long-term analgesic efficacy of opioids that is accompanied by the development of tolerance, opioid induced hyperalgesia (OIH), adverse side-effects, addiction, and opioid-related deaths [4,5]. New strategies to avoid the aversive effects of opioids while preserving their analgesic properties are therefore needed.

In this perspective, the serotonin 1 A receptor (5-HT_1A_) emerges as a promising candidate. 5-HT_1A_ is an inhibitory presynaptic autoreceptor on serotonergic neurons and is also expressed postsynaptically in terminal regions innervated by serotonergic neurons [6]. In animal studies, 5-HT_1A_ agonists have been reported to counteract opioid-induced hyperalgesia, opioid tolerance and to improve the analgesic potency of opioids while reducing their rewarding effects [6,7,8]. Contrary to opioids, a first order pronociceptive effect followed by an analgesic effect was documented for 5-HT_1A_ agonists, suggesting opposing effects between opioids and 5-HT_1A_ agonists [8]. Therefore, hypothetically, 5-HT_1A_/opioid interactions could be time-dependent with 5-HT_1A_ antagonists initially enhancing opioid analgesia [9,10] and 5-HT_1A_ agonists, having beneficial long-term effects when OIH has developed [6,7,8]. In agreement with this, a genetically inferred reduction of serotonergic signalling was associated with an increased analgesic response to the opioid drug fentanyl in healthy human subjects [11]. Furthermore, gene-to-gene interactions between the mu-opioid receptor (MOP) gene (*OPRM1*) and the serotonin transporter (*5-HTT*) or 5-HT_1A_ (*HTR1A*) genes had antagonistic effects on endogenous descending pain modulation in healthy subjects and in fibromyalgia patients [12].

In the human brain, high densities of 5-HT_1A_ [13] and MOP [14] have been reported in regions implicated in pain modulation, and high 5-HT_1A_ binding potential was associated with more efficient endogenous pain inhibition [15]. Moreover, significant positive associations were found between the serotonin and the opioid systems in networks known to regulate pain and mood, including the cingulate cortex, thalamus, dorsolateral prefrontal cortex, amygdala, and the left parietal cortex [16]. The exact mechanisms responsible for the physiological, pain-related interactions between the opioid and the serotonergic signalling systems are not known [6]. One possible mechanism is the opioid-induced activation of 5-HT_1A_–expressing glial cells through the Toll-like receptor 4 [17], as activated glia has been implicated in the development of OIH and opioid tolerance [18,19]. In accordance with this reasoning, extensive cortical glia activation was documented in patients suffering from fibromyalgia [20], a chronic pain syndrome with aberrations in cerebral opioid signalling [21]. An additional explanation might be the co-localization of MOP and 5-HT_1A_ receptors on the same neurons. In fact, Kishimoto et al. presented electrophysiological evidence of their co-localization on individual presynaptic GABAergic nerve terminals, and demonstrated that they synergistically inhibited GABA release in the periaqueductal gray (PAG), a structure that mediates opioid-based pain control [22]. In addition, the activation of GABA_A_ receptors in PAG projecting neurons was shown to have a net pronociceptive effect [23]. Further support for interactions between MOP and 5-HT_1A_ at the cellular level comes from a study showing that they can form functional heterodimers and that signalling of one receptor in the heterodimer is inhibited by the activation of the other [24]. We thus hypothesize that opioid induced heterodimerization of MOP and 5-HT_1A_ inactivates the receptors, which then become unable to inhibit GABA release and promote pronociceptive pathways.

The primary aim of this study was to challenge this hypothesis by quantitatively characterizing interactions between the MOP and 5-HT_1A_ receptors in live cells expressing near physiological levels of these receptors, and to assess the effects of commonly used non-peptide opioid drugs such as morphine, oxycodone, codeine, and fentanyl, on the extent of these interactions and their downstream effects. In particular, we have focused on intracellular Ca^2+^ levels and signalling dynamics, and on mitogen-activated protein kinases (MAPKs) p38 and the extracellular signal-regulated kinase (Erk1/2), both previously associated with the adverse effects of opioids [25,26].

## 2. Results

**Figure 1:**
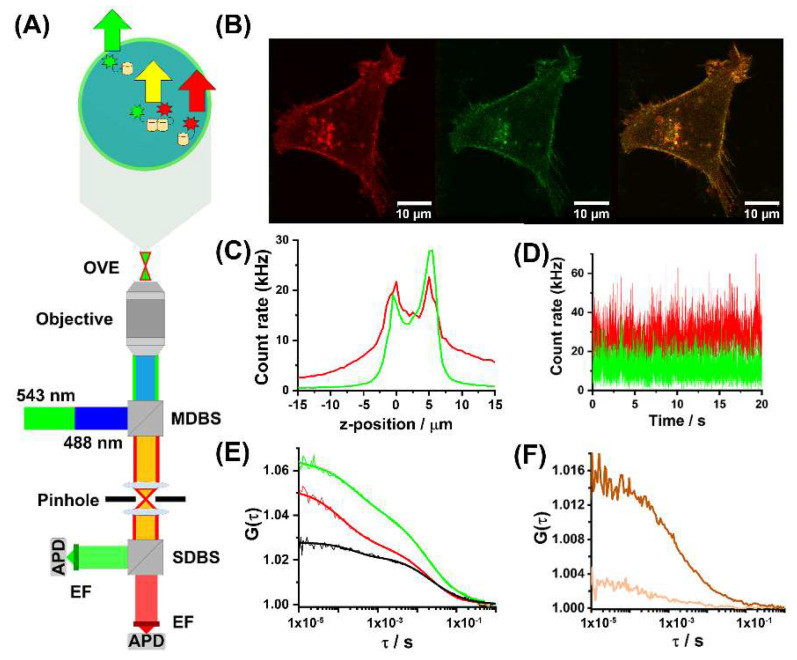
Fluorescence cross-correlation spectroscopy (FCCS). (**A**) Schematic presentation of the instrumental setup for dual colour CLSM imaging and FCCS. Incident laser light, 488 nm (blue) and 543 nm (green), is reflected by the main dichroic beam splitter (MDBS, 488/453/633) and focused by the objective into the sample. Fluorescence and scattered light are collected by the same objective and fluorescence is separated from the elastically scattered light by the MDBS. The fluorescence is spectrally separated by the secondary dichroic beam splitter (SDBS, 545) and further spectrally narrowed by emission filters (EF) before being recorded by avalanche photo diodes (APD) detectors. *Magnified insert:* Cross section through the observation volume element (OVE) in the radial (*xy*) plane in the sample. Fluctuations in fluorescence intensity are generated as fluorescently labelled molecules diffuse through the OVE (arrows). (**B**) CLSM image of a HEK293 cell genetically modified to stably express MOP-eGFP (green) and 5-HT_1A_-Tomato (red). Scale bar 10 µm. (**C**) Fluorescence intensity scan through a HEK293 cell expressing MOP-eGFP (green) and 5-HT_1A_-Tomato (red) in the axial (z-axis direction). The first peak in fluorescence intensity indicates the position of the basal (*z* = 0) and the second one the apical (*z* = 5 µm) plasma membrane of the same cell. Fluorescence intensity drops when the apical plasma membrane is crossed, as the OVE is now positioned in the surrounding cell culture medium. (**D**) Fluorescence intensity fluctuations recorded at the apical membrane of a HEK293 cell, originating from MOP-eGFP (green) and 5-HT_1A_-Tomato (red) lateral diffusion in the plasma membrane. (**E**) Representative auto- (green and red) and cross-correlation (black) curves recorded at the apical membrane of a HEK293 cell. (**F**) Cross-correlation curves recorded in live HEK293 cells expressing the positive (brown) and negative (champagne) control constructs. For detailed information see Section 4. Materials and Methods and Supplementary Materials, Section S1. Transfection, positive and negative control cells (Figures S1 and S2).

The effects of non-peptide opioids on the extent of interactions between the mu-opioid receptor fused with the enhanced Green Fluorescent Protein (MOP-eGFP) and the serotonin 1 A receptor fused with the red fluorescent protein Tomato (5-HT_1A_-Tomato) were examined in live cells using confocal laser scanning microscopy (CLSM) and fluorescence cross-correlation spectroscopy (FCCS). FCCS, a quantitative analytical method with single-molecule sensitivity, is succinctly described in Section 4. Materials and Methods. Primary data, temporal autocorrelation curves (tACCs) and cross-correlation curves (tCCC) acquired using FCCS are shown in Figure 1. Determination of the so-called relative cross-correlation amplitude (RCCA) and its use to assess the apparent dissociation constant is described in Section 4. Materials and Methods and in the Supplementary Materials, Section S2. Calculation of the apparent dissociation constant and S3. Relative Cross-Correlation Amplitude (RCCA) increased upon opioid treatment. Verification by switching FCCS. More information can also be found in [27].

### 2.1. Non-Peptide Opioids Potentiate MOP and 5-HT1A Heterodimerization to a Different Extent

**Figure 2:**
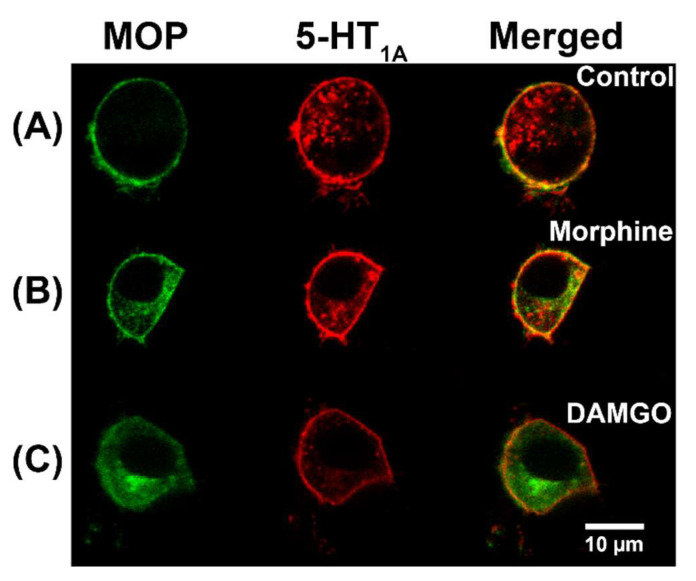
Non-peptide opioids neither induce MOP-eGFP nor MOP-eGFP-5-HT_1A_-Tomato heterodimers internalization, whereas the opioid peptide DAMGO induces strong MOP-eGFP internalization. CLSM images of live PC12 cells stably expressing MOP-eGFP (green) and 5-HT1A-Tomato (red). (**A**) Cultured under standard conditions, without opioid treatment (control). (**B**) Treated for 18 h with 750 nM morphine. (**C**) Treated for 18 h with 500 nM DAMGO. Scale bar 10 µm.

CLSM imaging showed clear co-localization of both receptors, MOP-eGFP (green) and 5-HT_1A_-Tomato (red), in the plasma membrane in both the HEK293 (Figure 1B) and the PC12 (Figure 2A) cells. It also showed that treatment with non-peptide opioids did not cause the internalization of individual receptors or of heterodimer receptor complexes (Figure 2B). This contrasts with the effects of treatment with the opioid peptide DAMGO, which promoted MOP internalization, but not the internalization of the heterodimer MOP-eGFP–5-HT_1A_-Tomato complex (Figure 2C).

**Figure 3:**
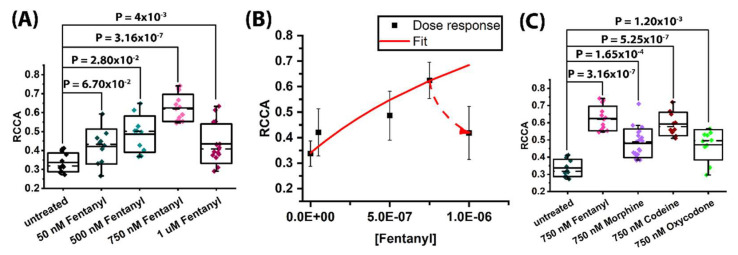
Opioids differ in their potency to induce heterodimer formation between MOP-eGFP and 5-HT_1A_-Tomato in HEK293 cells. (**A**) Fentanyl induces a dose-dependent increase in MOP-eGFP and 5-HT_1A_-Tomato receptor heterodimer formation in the concentration range 0 < c_Fentanyl_ < 750 nM. For fentanyl concentrations ≥ 1 µM, the extent of heterodimer formation drops significantly. (**B**) Fentanyl dose response curve calculated from the experimentally obtained RCCA values in A and the known concentrations of fentanyl. (**C**) 18 h treatment with equimolar concentrations of different opioids, c = 750 nM induces in cells expressing the same levels of MOP-eGFP and 5-HT_1A_-Tomato different extent of receptor heterodimer formation. Relative cross-correlation amplitude (RCCA), defined as the limiting value, when the lag time τ → 0, of the amplitude of the cross-correlation curve relative to the amplitude of the green autocorrelation curve, yields the number of dually-labelled, i.e., heterodimer receptor complexes (N_rg_) relative to the total number of the red labelled 5-HT_1A_-Tomato receptors (N_r_^total^ = N_r_ + N_rg_), where Nr is the number of unbound, single-labelled 5-HT_1A_-Tomato receptors, and N_rg_ is the number of double-labelled MOP-eGFP and 5-HT_1A_-Tomato complexes. To reduce the effect of noise and minimize the contribution of afterpulsing, the RCCAs values were calculated as an average value of five points, starting with the value at the lag time of 10 µs to the lag time of 50 µs. In the box-and-whisker plot, the solid line shows the mean value, the dashed line shows the median, box represents the standard deviation, and the whiskers give the 5-95 percentiles. Statistical analysis: a Student’s *t*-test was used to determine whether the difference between the mean values measured in untreated and treated cells, or in cells treated with different opioids, are significantly different from each other. The results are reported using a two-tailed *p*-value (*p*). The Benjamini–Hochberg method to control the false discovery rate (FDR) in sequential modified Bonferroni correction for multiple hypothesis testing showed that at an FDR value of 5%, *p* ≤ 0.012 was statistically significant.

For FCCS analysis, data collected on cells expressing similar (within the experimental error of FCS measurements) receptor levels, N_MOP_ = (27 ± 6) and N_5-HT1A_ = (25 ± 3), were compared. At these expression levels, corresponding to concentrations: c_MOP_ = (320 ± 70) nM and c_5-HT1A_ = (300 ± 40) nM, FCCS analysis showed that MOP-eGFP and 5-HT_1A_-Tomato receptors not only co-localized in the plasma membrane, but also formed heterodimers, as evidenced by tCCCs (Figure 1E, black). FCCS showed that in untreated cells about 33% (RCCA = 0.33) of the 5-HT_1A_-Tomato receptors are bound in heterodimer complexes with MOP-eGFP (Figure 3A). Based on this, the apparent dissociation constant for a heterodimer receptor complex of MOP-eGFP–5-HT_1A_-Tomato with a 1:1 stoichiometry was estimated to be $K_{d}^{\mathrm{app}}$ = (440 ± 70) nM.

Moreover, FCCS showed that treatment with different concentrations of fentanyl increased the fraction of 5-HT_1A_-Tomato receptors in heterodimer complexes with MOP-eGFP (Figure 3A,B). For fentanyl, the number of heterodimer receptor-receptor complexes increased in a dose dependent manner, as evident from the increase in RCCA from ${\mathrm{RCCA}}_{\mathrm{Fentanyl}}^{50\;\mathrm{nM}}$ = 0.42 ± 0.09, which was not significantly different from the RCCA value measured in untreated cells (*p* = 0.067), to ${\mathrm{RCCA}}_{\mathrm{Fentanyl}}^{500\;\mathrm{nM}}$ = 0.49 ± 0.09 (*p* = 0.028) in cells treated with 500 nM fentanyl, and ${\mathrm{RCCA}}_{\mathrm{Fentanyl}}^{750\;\mathrm{nM}}$ = 0.62 ± 0.07 (*p* = 3.16 × 10^−7^) in cells treated with 750 nM fentanyl. From the experimentally determined concentration of heterodimer complexes and the known concentration of fentanyl, the effect of fentanyl on the extent of MOP-eGFP and 5-HT_1A_-Tomato heterodimerization could be quantified (Figure 3B, solid red line).

By applying the standard mathematical formalism of ligand binding assays in the absence of competing reactions [28], and considering the concentration of heterodimer complexes as an dependent variable and the concentration of fentanyl as an independent variable, the concentration of fentanyl at which the number of heterodimer complexes would be doubled was determined to be (1.90 ± 0.05) µM. Unexpectedly, treatment with such high fentanyl concentrations showed a decrease, rather than the expected increase in the concentration of heterodimer complexes (Figure 3B, dashed red line) and the RCCA decreased to 0.45 (SD = 0.11, *p* = 0.004). This suggested that other processes, such as receptor homodimer formation and/or higher-order receptor heterooligomer formation [29,30] and/or desensitization or feedback processes [30] may occur at high fentanyl concentrations. Finally, it may also happen that fentanyl at such high concentrations may be toxic to cells [31], but we have not observed any such indication.

Due to this, concentrations higher than 750 nM were not investigated, and this concentration was selected in further studies to compare the effects of different opioids. FCCS showed that for treatment with 750 nM fentanyl, the RCCA was ${\mathrm{RCCA}}_{\mathrm{Fentanyl}}^{750\;\mathrm{nM}}$ = 0.62 ± 0.07, which was significantly different from the RCCA value measured in untreated cells (*p* = 3.16 × 10^−7^); ${\mathrm{RCCA}}_{\mathrm{Morphine}}^{750\;\mathrm{nM}}$ = 0.47 ± 0.08 (*p* = 1.65 × 10^−4^); ${\mathrm{RCCA}}_{\mathrm{Codeine}}^{750\;\mathrm{nM}}$ = 0.59 ± 0.07 (*p* = 5.25 × 10^−7^) and ${\mathrm{RCCA}}_{\mathrm{Oxycodone}}^{750\;\mathrm{nM}}$ = 0.47 ± 0.09 (*p* = 0.0117) (Figure 3C). Moreover, the RCCA value measured for cells treated with fentanyl was significantly higher than that measured in cells treated with equimolar concentrations of morphine (*p* = 2.48 × 10^−4^) or oxycodone (*p* = 6.99 × 10^−4^), but not significantly higher than that for codeine (*p* = 0.24). The difference in RCCA values measured in cells treated with codeine was significantly higher than that in cells treated by morphine (*p* = 3.66 × 10^−3^). Based on these measurements and using Equation (6), the apparent dissociation constants for the MOP-eGFP–5-HT_1A_-Tomato heterodimer complex in the presence of equimolar concentrations (750 nM) of different non-peptide opioids could be estimated: $K_{d,\;\mathrm{Fentanyl}}^{\mathrm{app}}$ = (80 ± 70) nM, $K_{d,\mathrm{Morphine}}^{\mathrm{app}}$ = (200 ± 70) nM, $K_{d,\;\mathrm{Codeine}}^{\mathrm{app}}$ = (100 ± 70) nM and $K_{d,\;\mathrm{Oxycodone}}^{\mathrm{app}}$ = (200 ± 70) nM. Likewise, the apparent heterodimer dissociation constants in the presence of different concentrations of fentanyl were determined to be: $K_{d,\;50\;\mathrm{nM}\;\mathrm{Fentanyl}}^{\mathrm{app}}$ = (260 ± 70) nM, $K_{d,\;500\;\mathrm{nM}\;\mathrm{Fentanyl}}^{\mathrm{app}}$ = (180 ± 70) nM, $K_{d,\;750\;\mathrm{nM}\;\mathrm{Fentanyl}}^{\mathrm{app}}$ = (80 ± 70) nM, and $K_{d,\;1\;µM\;\mathrm{Fentanyl}}^{\mathrm{app}}$ = (220 ± 70) nM.

### 2.2. Non-Peptide Opioids Increase to a Different Extent the Brightness of eGFP and Tomato

Prolonged treatment with non-peptide opioids increased eGFP brightness, as evident from the measured counts per second per molecule (CPM). In untreated cells, average eGFP brightness was ${\mathrm{CPM}}_{\mathrm{Untreated}}^{\mathrm{eGFP}}$ = (1.1 ± 0.3) kHz. In treated cells, eGFP brightness nearly doubled, showing statistically significant difference for all treatments: ${\mathrm{CPM}}_{\mathrm{Fentanyl}}^{\mathrm{eGFP}}$ = (1.9 ± 0.7) kHz (*p* = 0.015), ${\mathrm{CPM}}_{\mathrm{Morphine}}^{\mathrm{eGFP}}$ = (2.0 ± 0.5) kHz (*p* = 9.6 × 10^−3^), ${\mathrm{CPM}}_{\mathrm{Codeine}}^{\mathrm{eGFP}}$ = (1.9 ± 0.5) kHz (*p* = 5.8 × 10^−3^), and ${\mathrm{CPM}}_{\mathrm{Oxycodone}}^{\mathrm{eGFP}}$ = (1.8 ± 0.7) kHz (*p* = 0.027). Interestingly, an increase in Tomato brightness was also observed in cells treated with 750 nM fentanyl or morphine, but not in cells treated with codeine or oxycodone. However, the increase in Tomato brightness was not as pronounced as for eGFP, and changed from ${\mathrm{CPM}}_{\mathrm{Untreated}}^{\mathrm{Tomato}}$ = (0.8 ± 0.2) kHz in untreated cells to: ${\mathrm{CPM}}_{\mathrm{Fentanyl}}^{\mathrm{Tomato}}$ = (1.1 ± 0.3) kHz (*p* = 0.021) for treatment with 750 nM fentanyl; ${\mathrm{CPM}}_{\mathrm{Morphine}}^{\mathrm{Tomato}}$ = (1.3 ± 0.3) kHz (*p* = 3.0 × 10^−3^) for treatment with 750 nM morphine, whereas it remained unchanged (within the limits of the experimental error) for treatment with 750 nM codeine, ${\mathrm{CPM}}_{\mathrm{Codeine}}^{\mathrm{Tomato}}$ = (1.0 ± 0.3) kHz (*p* = 0.20), or 750 nM oxycodone, ${\mathrm{CPM}}_{\mathrm{Oxycodone}}^{\mathrm{Tomato}}$ = (0.9 ± 0.3) kHz (*p* = 0.12). While we do not know why the brightness of fluorescence reporters has changed following treatment with non-peptide opioids, two processes can independently and jointly cause such effects, receptor homodimerization and/or alteration of fluorescence lifetime due to environmental changes. However, to discern the contribution of one effect from the other, a stringent number and brightness analysis and fluorescence lifetime measurements would be needed. We reflect on this in more detail in Section 3.

### 2.3. Non-Peptide Opioids Elicit Different Intracellular Ca^2+^ Signalling Dynamics

**Figure 4:**
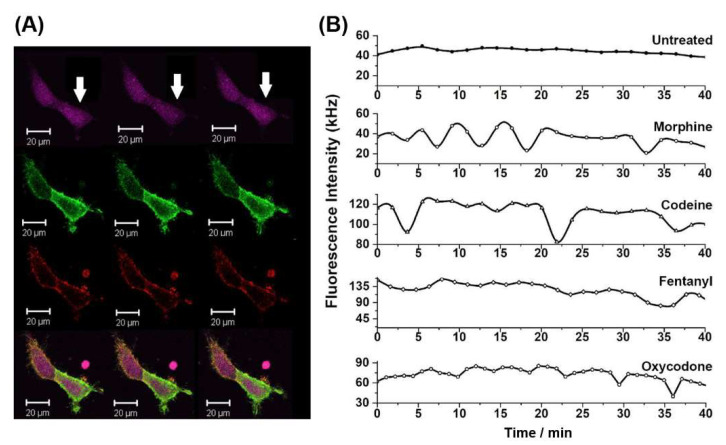
Stimulation with non-peptide opioids causes different intracellular Ca^2+^ signalling dynamics in HEK293 cells stably expressing MOP-eGFP and 5-HT_1A_-Tomato. (**A**) CLSM time-lapse imaging of Ca^2+^ levels (Fura Red, dark violet) in HEK293 cells expressing MOP-eGFP (green) and 5-HT_1A_-Tomato (red) after 30 min treatment with 750 nM oxycodone. White arrows indicate oscillatory changes in Fura Red fluorescence intensity, where a transient decrease in fluorescence intensity reflects an increase in the concentration of Ca^2+^ ions. (**B**) Fluctuations in Fura Red fluorescence intensity over time following treatment of HEK293 cells expressing MOP-eGFP and 5-HT_1A_-Tomato with equimolar concentrations of different opioids.

Time-lapse CLSM imaging of intracellular Ca^2+^ levels using the cell-permeant Fura Red ratiometric dye (Figure 4A), showed that stimulation with equimolar concentrations of different non-peptide opioids acutely induced different changes in the intracellular Ca^2+^ levels in HEK293 cells expressing MOP-eGFP and 5-HT_1A_-Tomato (Figure 4B). In untreated cells, stationary state intracellular Ca^2+^ levels were observed. Following the addition of 750 nM morphine, the stationary state appeared to have lost its stability and sinusoidal oscillations in Ca^2+^ levels with smoothly increasing amplitudes and a period of about 5 min, emerged. Treatment with 750 nM codeine also induced oscillations in intracellular Ca^2+^ levels. However, these oscillations showed features of so-called relaxation oscillations [32], which are characterized by a relatively long relaxation period during which the system remained in a stationary state, alternating with a short period in which the abrupt decrease in fluorescence intensity, i.e., the increase in intracellular Ca^2+^ level was observed. Treatment with 750 nM fentanyl did not cause any oscillations in intracellular Ca^2+^ levels, but a four-fold increase in Fura Red fluorescence intensity was noted, indicating that intracellular Ca^2+^ levels decreased markedly following the addition of fentanyl. Finally, treatment with 750 nM oxycodone induced small-amplitude relaxation oscillations with gradually increasing amplitudes over a period of about 5 min. Of note, while the time series shown in Figure 4 was recorded in individual cells, the dynamic behaviour is representative, as it is most often encountered in the analysed population of cells.

### 2.4. Non-Peptide Opioids Differ in the Extent to Which They Activate Major Signal Transduction Pathways

**Figure 5:**
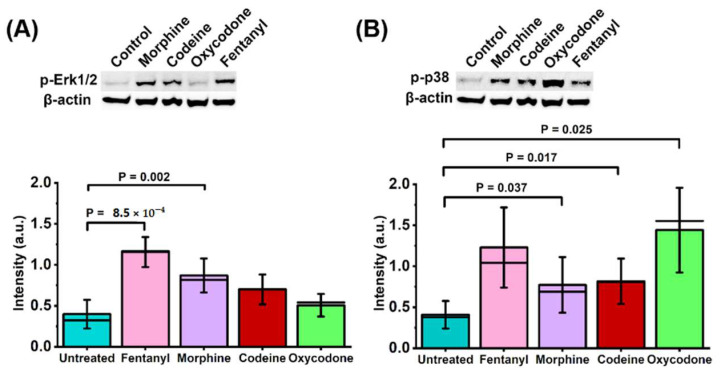
Opioids differ in their capacity to activate extracellular signal-regulated kinase (ERK) and p38 mitogen-activated protein kinase (MAPK) signalling pathways in HEK293 cells stably expressing MOP-eGFP and 5-HT_1A_-Tomato. Top: HRP chemiluminescence images of western blotting membranes showing different levels p-ERK1/2 (**A**) and p-p38 (**B**) following 18 h treatment using equimolar concentrations of different opioids. Bottom: Protein level of phosphorylated-Erk1/2 relative to β-actin (**A**) and the protein level of phosphorylated-p38 relative to β-actin (**B**), following 18 h treatment with 750 nM of morphine (red), codeine (green), oxycodone (blue) or fentanyl (magenta), as compared to untreated cells (grey). Statistical analysis: Paired *t*-test. Experiments were carried out in triplicate (see Supplementary Materials, Section S4. Western blotting (Figure S5)).

In order to assess the downstream effects of non-peptide opioids in HEK293 cells expressing MOP-eGFP and 5-HT_1A_-Tomato, phosphorylation of MAPKs ERK1/2 and p38 was probed because MOP activation was shown to trigger the phosphorylation of both ERK1/2 [33] and p38 [34]. Western blot analysis showed an increase in phosphorylated ERK1/2 and p38 in cells that had been treated with 750 nM of morphine, oxycodone, codeine, or fentanyl when compared to untreated cells (Figure 5).

Fentanyl elicited the strongest ERK1/2 activation (mean = 1.156, SD = 0.183, *p* = 8.52 × 10^−4^), unlike oxycodone (mean = 0.506, SD = 0.139, N.S). In contrast, oxycodone elicited the strongest p38 activation (mean = 1.441, SD = 0.517, *p* = 0.025), while the effects of fentanyl, morphine, and codeine were similar. Interestingly, LC-MS/MS metabolite analysis indicated that these effects are likely attributed to the primary non-peptide opioid compounds in their own right, as there were no common opioid metabolites detected either in the cell culture medium or in the cell lysate (Supplementary Materials, Section S6, Table S2).

## 3. Discussion

Advanced fluorescence microscopy-based techniques allow us to quantitatively characterize molecular interactions in live cells and bring about new understanding of dynamical processes that underlie complex biological functions [35,36,37]. They also enable us to test with unprecedented precision new mechanistic hypotheses. In this study, FCCS, a quantitative time-resolved analytical method with single-molecule sensitivity, was used to examine in live cells the hypothesis that prolonged exposure to non-peptide opioids promotes heterodimer formation between MOP and 5-HT_1A_. This hypothesis, derived from preclinical [6,7,8] and clinical studies [11,12,20], further asserts that altered cellular signalling due to receptor heterodimer formation may contribute to neuroplastic changes that, eventually, lead to sensitization of pronociceptive pathways at the organism level.

To test the initial statement in this hypothesis, FCCS was used to quantitatively characterize in live cells interactions between MOP and 5-HT_1A_ receptors and the effects of some of the most commonly used non-peptide opioid drugs: morphine, oxycodone, codeine and fentanyl, on the extent of these interactions. The CLSM imaging, biochemical assays and LC-MS/MS were used to assess the downstream consequences of these interactions. The most important results are summarized in Table 1 and discussed below.

We found that MOP and 5-HT_1A_ receptors associate in the plasma membrane (Figure 1B, Figure 2A and Figure 3A), building heterodimer complexes characterized by an apparent dissociation constant, $K_{d}^{\mathrm{app}}$ = (440 ± 70) nM. This result, obtained nondestructively in live cells, confirms the findings by Cussac et al. who have shown using co-immunoprecipitation and Bioluminescence Resonance Energy Transfer (BRET) that functional MOP and 5-HT_1A_ heterodimers are formed in overexpressing cells [24]. We have verified this finding in cells expressing physiologically relevant levels of the investigated receptors and determined the apparent dissociation constants for MOP–5-HT_1A_ heterodimers in live cells, $K_{d}^{app}$ = (440 ± 70) nM. Moreover, in agreement with the results obtained by Cussac et al. [24], we have also observed that DAMGO induces prominent MOP internalization but not the internalization of MOP–5-HT_1A_ heterodimer complexes, whereas the non-peptide opioids did not cause internalization, neither of individual receptors, nor of heterodimer MOP–5-HT_1A_ complexes (Figure 2).

Prolonged exposure to all opioids tested facilitated heterodimer formation between MOP and 5-HT_1A_ receptors, albeit to a different extent (Figure 3), differently altered intracellular Ca^2+^ levels and signalling dynamics (Figure 4) and activated ERK1/2 and p38 signal transduction pathways to a different extent (Figure 5). Fentanyl, the most potent off all non-peptide opioids tested here, exhibited in the concentration range 50–750 nM, a dose-dependent effect on MOP–5-HT_1A_ heterodimer formation (Figure 3A,B) and stabilized significantly the heterodimer complexes, as evident from the five-fold decrease in the apparent dissociation constant from $K_{d}^{\mathrm{app}}$ = (440 ± 70) nM in untreated cells, to $K_{d,\;\mathrm{Fentanyl}}^{\mathrm{app}}$ = (80 ± 70) nM in cells treated with 750 nM fentanyl. It also elicited the highest activation of the ERK1/2 and a comparably strong activation of the p38 (Figure 5). Finally, fentanyl caused an acute decrease in Ca^2+^ levels, as evident from the pronounced increase in Fura Red fluorescence (Figure 4, Table 1), which is in line with previously reported findings [38]. In contrast, oxycodone elicited the weakest stabilizing effect on MOP–5-HT_1A_ heterodimer formation, as evident from the two-fold reduction in $K_{d,\;\mathrm{Oxycodone}}^{\mathrm{app}}$ = (200 ± 70) nM (Figure 3C) and elicited the highest activation of the p38 (Figure 5B), while causing an insignificant activation of the ERK1/2 (Figure 5A, Table 1). Treatment with 750 nM oxycodone did not significantly affect Ca^2+^ signalling dynamics, although a small reduction in Ca^2+^ level and the appearance of small-amplitude relaxation oscillations were noted. The very strong activation of the p38 observed in our study is in line with recent findings in rats showing increased p38 activity during chronic oxycodone exposure [39]. p38 activation may also be relevant for the aversive, addictive effects of oxycodone—p38 activation was shown to underlie opioid reward behaviour in mice [40] and the kappa opioid receptor (KOP)-induced p38 activation has been shown to reinstate drug seeking behavior in mice [41]. Based on this, a recent study argued that the addictive qualities of oxycodone outweighed its benefits as a prescription drug [42].

Morphine and codeine showed significant differences in their potency to stabilize MOP–5-HT_1A_ heterodimer complexes, with codeine eliciting a higher stabilizing effect than morphine, $K_{d,\mathrm{Morphine}}^{\mathrm{app}}$ = (200 ± 70) nM and $K_{d,\;\mathrm{Codeine}}^{\mathrm{app}}$ = (100 ± 70) nM (Figure 3C, Table 1). Codeine also elicited more dramatic effects on intracellular Ca^2+^ signalling, reducing to a larger extent intracellular Ca^2+^ levels and causing more dramatic changes in Ca^2+^ signalling dynamics than morphine (Figure 4B). However, they activated the ERK1/2 and p38 signalling pathways to a similar extent. The unexpected observation that codeine more strongly stabilized MOP and 5-HT_1A_ heterodimer complexes than morphine is contrary to the general view that codeine is an inactive prodrug with a low affinity for MOP, the effect of which is obtained first after its metabolic conversion to morphine [43,44,45] and dihydrocodeine-6-O-gluconoride [46,47]. To interrogate this further, an LC-MS/MS analysis was deployed. The LC-MS/MS showed that the concentration of codeine metabolites in the cell culture medium and the cell lysate, if present at all, is below the detection limit of the applied method (Supplementary Materials, Section S6, Table S1). This finding is in line with the fact that HEK293 cells do not express the CYP2D6 gene (https://www.proteinatlas.org/ENSG00000100197-CYP2D6/cell, 3 February 2022), which is crucial for metabolizing codeine [48]. Taken together, our data indicate that codeine is an active compound in its own right. Recent studies showed that codeine has a 6-fold higher permeability and crosses the plasma membrane faster than morphine [49], which could potentially explain the strong response elicited in our cell model. This finding suggests that the pharmacodynamics of codeine is not yet fully elucidated and warrants further studies.

Moreover, FCCS analysis revealed that all non-opioid peptides tested nearly doubled eGFP brightness, while Tomato brightness was not affected to the same extent and treatment with oxycodone and codeine did not alter Tomato brightness (Table 1). The following processes: homodimerization of MOP and, to a lesser extent, of 5-HT_1A_; homo- and heterooligomerization of MOP and 5-HT_1A_; and changes in fluorescence lifetime of eGFP and Tomato due to intracellular environment changes caused by signal transduction, can independently or jointly increase the brightness of eGFP/Tomato. Further studies are, however, needed to distinguish the contribution of these possible mechanisms. Most notably, stringent number and brightness analysis and fluorescence lifetime measurements would be needed to discern the contribution of one effect from the other. Having said this, we point out that changes in brightness consistent with the presence of higher order oligomers were not observed.

The possibility to quantitatively characterize MOP/5-HT_1A_ interactions in live cells is a significant achievement of great general interest—the stability of G protein-coupled receptor (GPCR) homo/heterodimer complexes is measured in live cells for a handful of GPCRs, see for example [34,35,36,50], even though it is well recognized that numerous GPCRs form homo- and heterodimers and that these interactions are important targets for drug development [51]. However, some limitations of our approach are inevitably present and warrant further discussion. Most notably, FCS/FCCS cannot detect endogenous nonfluorescent receptors, receptor constructs with irreversibly photobleached fluorophores or with fluorophores residing for various reasons in dark states. This affects the actual value of the apparent dissociation constants. However, in the context of our study, this limitation does not affect the conclusions of our work, since relative differences are analysed.

Another limitation of our study is that the work was performed using transfected cells that express the proteins of interest through powerful promoters, which may lead to artefacts due to over-expression. To mitigate this risk, we have generated stably transformed cell lines—it is commonly known that stably transformed cells do not yield as high expression as transiently transfected cells. Besides, we have selected for our analysis cells expressing low levels of MOP-eGFP and 5-HT_1A_-Tomato—the average number of molecules in the OVE of N_MOP_ = (27 ± 6) and N_5-HT1A_ = (25 ± 3) corresponds to a surface density of about (130 ± 10) molecules/µm^2^. For comparison, many studies of GPCRs class A show that the average surface density of endogenous GPCRs is typically low, <5 molecules/µm^2^, in healthy but increase severalfold in disease states—a recent study showed endogenous MOP levels of 4 molecules/µm^2^ [52]. However, as cautioned by the authors, one needs to bear in mind that this value may be underestimated due to low antibody binding efficiency—theoretical studies show that at GPCRs surface densities < 5 molecules/µm^2^ the receptors may be too far apart from each other to allow for the efficient build-up of Gβγ to concentrations needed to modulate the activity of other intracellular proteins and show that G protein signalling occurs within nanodomains where the local density of GPCRs is easily > 50 molecules/µm^2^ [53].

Furthermore, an important limitation of our study is that the effects of one concentration of non-peptide opioids is tested. A dose-response analysis is needed to characterize cellular responses to varied amounts of the selected non-peptide opioids and stringent control experiments are needed to examine to what extent the observed effects are mediated via the monomeric fraction of the receptor pool and what the contribution of the receptor heterodimer is. In the light of our work, it is important to point out that while the affinity of the tested non-peptide opioids for binding to MOP is in the range 1–750 nM [54] and to 5-HT_1A_ in the 2–20 µM range [55], pharmacologically relevant concentrations of non-peptide opioids are considerably higher [56]. For example, in opioid-naive postoperative patients, an analgesic effect of fentanyl is achieved at the lowest blood plasma fentanyl concentration levels of about 1.8–4.4 nM (0.6–1.5 ng/mL) [57]. However, much higher concentrations were measured in cancer patients treated for pain; on average 530 nM (178 ng/mL) [56,58]. The concentrations used in our study are therefore in the pharmacologically relevant range. Moreover, in this study we chose to study equimolar concentrations of opioids, rather than equipotent concentrations. Although there are several conversion tables for opioid potency, they are perceived as unreliable [59]. The few studies that have addressed opioid equianalgesic dose/potency ratios are heterogeneous with respect to size, subjects, specific aims, settings, and study method [60]. Thus, Rennick et al. have concluded from their findings that there is no true universal way to accurately perform equianalgesic conversions for opioids [60]. Given that the aim of our work was to assess the effect of non-peptide opioids on the extent of MOP-eGFP and 5-HT_1A_-Tomato association in live cells, the study design where cells with similar receptor surface density levels are used and the effects of equimolar concentrations of non-peptide opioids are compared is correct. It may, however, be interesting to examine in the future the effect of equipotent concentrations of non-peptide opioids, determined with regard to a quantifiable effect, such as the ability to alter intracellular Ca^2+^, ERK1/2 or p38 phosphorylation levels.

Furthermore, the treatment time length is an important variable. We have chosen 18 h, taking into consideration the cell doubling time, which is under the condition of our experiments ~36 h for HEK293 cells and ~72 h for PC12 cells. In this way, the cells were exposed to treatment for a considerable fraction, 0.25–0.50, of their cycle time and the effect of the number of divisions during the course of an assay is small [61]. In future studies it may, however, be interesting to examine the effect of treatment time length on MOP-eGFP and 5-HT_1A_-Tomato association in live cells in order to understand the relevance of this phenomenon in acute vs chronic treatment with non-peptide opioids.

Finally, we have not used in our study antagonists of MOP and 5-HT_1A_ receptors to block effects mediated via monomeric receptors. Consequently, we cannot discern to what extent Ca^2+^ level and dynamics, and ERK1/2 or p38 phosphorylation levels change via heterodimer-mediated pathways and whether these effects can also be blocked by the selective antagonists of MOP and 5-HT_1A_.

## 4. Materials and Methods

### 4.1. Cell Culture and Transfection

Human embryonic kidney (HEK293) and rat phaeochromocytoma (PC12) cell lines (American Type Culture Collection (ATCC)), were used because they are capable of post-translational folding and modifications required to express MOP and 5-HT_1A_ [62,63,64]. The experiments were first performed in HEK293 cells, and key findings were validated in PC12 cells. The HEK293 and PC12 cells were stably transformed to simultaneously express the human MOP receptor genetically fused at the C-terminus with the enhanced Green Fluorescent Protein (MOP-eGFP) and the human 5-HT_1A_ receptor genetically fused at the C-terminus with the Tomato Red Fluorescent Protein (5-HT_1A_-Tomato). Both constructs were cloned into the pBudCE4.1 vector (Thermo Fisher, Munich, Germany), with the MOP-eGFP gene being expressed under the control of the hEF-1 promoter (KpnXho) and 5-HT_1A_-Tomato gene under the control of the CMV promoter (HindXba). For details, see Supplementary Materials (Section S1, Figure S1A).

For cultivation, untransformed and stably transformed HEK293 and PC12 cells were cultured in collagen coated T25 flasks (Sarsted) at 37 °C in a humidified atmosphere containing 5% CO_2_. HEK293 cells were cultured in DMEM medium supplemented with 10% Fetal Bovine Serum (FBS), 100 U/mL penicillin and 100 μg/mL streptomycin (PenStrep). For PC12 cells, the RPMI 1640 medium supplemented with 10% Horse Serum (HS) and 5% FBS, 100 U/mL penicillin and 100 μg/mL streptomycin was used. All cell culture reagents were from Invitrogen, Stockholm, Sweden.

For generating the stably transformed cell lines, the HEK293 and PC12 cells were grown to 70% confluence in 8-well chambered cover slides (Nalge Nunc International, Rochester, NY, USA) and transfected using Lipofectamine 2000 (Invitrogen), following the transfection protocol provided by the manufacturer. Stably expressing cell lines were isolated through selection using culture medium supplemented with phleomycin D1 antibiotic (0.4 mg/mL, Thermo Fisher). Positive and negative control cells were cultured and transfected in the same way. For details, see Supplementary Materials (Section S1, Figure S1A,B). The functionality of MOP-eGFP and 5-HT_1A_-Tomato receptors was validated by assessing how treatment with the selected agonists morphine, serotonin or buspirone or their combination: morphine and serotonin, or morphine and buspirone, affects phosphorylation of Erk1/2 and p38 MAPKs. The data show that all tested compounds and their combination increase the protein levels of p-ERK1/2 and p-p38 as compared to their levels in untreated cells (Section S1, Figure S1C).

### 4.2. Confocal Laser Scanning Microscopy (CLSM) Imaging and Fluorescence Correlation and Cross-Correlation Spectroscopy (FCS/FCCS)

The CLSM imaging and FCS/FCCS were performed using an individually modified ConfoCor 3 system (Carl Zeiss, Jena, Germany), as previously described [65,66]. Briefly, the system comprises an inverted microscope for transmitted light and epifluorescence (Axiovert 200 M); a VIS-laser module housing the Ar/ArKr (458, 477, 488 and 514 nm), HeNe 543 nm and HeNe 633 nm lasers; a scanning module LSM 510 META modified to enable imaging using silicon avalanche photodiodes (SPCM-AQR-1X, PerkinElmer, Waltham, MA, USA) in order to allow studies of cells expressing low levels of the proteins of interest; and an FCS/FCCS module with two detection channels. The C-Apochromat 40×/1.2 W UV-VIS-IR objective was used throughout. A stage incubator consisting of a heated microscope stage (Heating insert P), incubator box (Incubator S), atmosphere-controller (CTI-Controller 3700) and a temperature regulator (Temp control 37-2 digital), was used to maintain the cells at 37.0 °C and supply them with heated humidified air containing 5.0% CO_2_. The temperature and CO_2_ levels were continuously monitored and regulated via a digital feedback control algorithm, allowing temperature control within ±0.2 °C and atmosphere control within ±0.1% CO_2_.

The CLSM images were acquired in a sequential, i.e., dual track mode, one channel at a time. The eGFP fluorescence was excited using the 488 nm line of the Ar/ArKr laser. A band pass 505–530 nm emission filter was used to spectrally narrow the emitted fluorescence. Tomato fluorescence was excited using the 543 nm HeNe laser, and a long pass 580 nm emission filter was used to collect the emitted fluorescence. Incident and emitted light were separated using the main dichroic beam splitter HFT 488/543/633. The eGFP and Tomato fluorescence were separated using a secondary dichroic beam splitter NFT 545 (Figure 1A). Images were acquired without averaging, using a pixel dwell time of 51.2 µs and a 512 × 512 pixels format (Figure 1B).

The optical setup for FCCS was the same as for CLSM imaging described above. Fluorescence intensity fluctuations were recorded at the apical plasma membrane of live cells identified by an axial fluorescence intensity scan (Figure 1C). Time series were collected in an array of 10 consecutive measurements, each measurement lasting 20 s (Figure 1D).

### 4.3. Brief Background on FCS/FCCS

Fluorescence cross-correlation spectroscopy (FCCS) is a dual color variant of fluorescence correlation spectroscopy (FCS). The FCS measures with sub-microsecond temporal resolution spontaneous fluctuations in fluorescence intensity around a steady state to extract quantitative information about the concentration and diffusion/size of fluorescent molecules [67,68,69]. The FCS is well suited for biological applications, as it is non-destructive and allows quantitative measurements to be performed in sub-cellular compartments [70]. The fluctuations in fluorescence intensity are recorded in a very small observation volume element (OVE) that is typically about V_OVE_ = 0.1–2 fL ((0.1–2) × 10^−15^ L). The OVE is generated by tightly focusing the incident laser light into the sample using a high numerical aperture objective. Fluorescence is collected through the same objective, and the volume from which fluorescence is being collected is reduced by placing a pinhole in the optically conjugate plane in front of the detector [69,71]. The spontaneous diffusion of fluorescent molecules in and out of the OVE gives rise to fluctuations in fluorescence intensity. The size and volume of the OVE is specific for each instrument and is determined in calibration experiments using a reference molecule with a known diffusion coefficient, such as Rhodamine 6G (Rh6G). Using a standard 10 nM Rh6G solution, and following the procedure described in detail in [71], the OVE volume in our system was determined to be V_OVE_ = 0.2 fL [68]. For a quick estimate of the concentration, note that for a 10 nM solution and V_OVE_ = 0.17 fL, the average number of molecules in the OVE (N) is N = 1 [71,72].

The FCS/FCCS works best at low, sub-micromolar concentrations [71], where the signal from a bright fluorescent molecule generates a substantial increase in fluorescence intensity that is well above the background signal from the surrounding molecules. From the recorded fluorescence intensity fluctuations, which are generated by the translational diffusion of fluorescent molecules, one can extract the average number of molecules in the OVE (N), i.e., their concentration and their average translational diffusion time (τ_D_), which is defined by the diffusion coefficient (D), i.e., the size of the molecule (Stokes-Einstein equation). To extract this information from fluorescence intensity fluctuation analysis, the most commonly employed method, which is also used here, is temporal autocorrelation analysis. In temporal autocorrelation analysis, the signal is compared to a copy of itself delayed for a certain lag time (τ) using the autocorrelation function:

(1) $$G\left(\tau \right)=1+\frac{\langle \delta F\left(t\right)\cdot \delta F\left(t+\tau \right)\rangle }{{\langle F\left(t\right)\rangle }^{2}}$$

In Equation (1), chevron brackets denote average values of the analyzed variables over time, and fluorescence intensity fluctuation (δ(F(t)) is the deviation of the fluorescence intensity at time t (F(t)) from the mean fluorescence intensity (〈F(t)〉), δF(t) = F(t) − 〈F(t)〉. Accordingly, δF(t + τ) = F(t + τ) − 〈F(t)〉. When the fluctuations are not random, temporal autocorrelation analysis yields a temporal autocorrelation curve (tACC). The tACC is characterized by a maximal limiting value of G(τ) as τ → 0 and decreases to the value G(τ) = 1 at long lag times, indicating that correlation between the fluorescence intensities is being lost (Figure 1E, green and red). If there is only one process that gives rise to fluorescence intensity fluctuations, the tACC shows one inflection point, that is, one characteristic decay time. If there are more processes giving rise to fluorescence intensity fluctuations, which occur at different time scales, the tACC assumes a more complex shape with more than one characteristic decay time (Figure 1E, green and red). The zero-lag amplitude of the tACC, (G_0_ = G(0) − 1) provides information about the concentration of fluorescent molecules as it equals the inverse average number of molecules in the OVE (1/N). Thus, the amplitude of the tACC decreases when N increases. The characteristic decay time of the tACC gives information about the rates at which processes that give rise to the fluorescence intensity fluctuations occur. When fluorescence intensity fluctuations are generated by molecular diffusion, the characteristic decay time of the tACC reflects the average time it takes for a molecule to cross through the OVE by translational diffusion.

For dual colour FCCS, two spectrally distinct fluorescent molecules, such as eGFP and Tomato, are used to render the molecules of interest visible. Fluorescence intensity fluctuations are then simultaneously recorded for both fluorophores using overlaying excitation pathways, but separate detector pathways (Figure 1A). The fluorescence intensity fluctuations observed in FCCS (Figure 1D) are subjected to temporal auto- and cross-correlation analysis. This generates two tACCs, one for each fluorophore (Figure 1E, red and green) and, when the molecules of interest bind, one temporal cross-correlation curve (tCCC; Figure 1E, black), which reflects the population of dually labelled molecules diffusing as one [36,73,74]. As in FCS, the amplitudes of the individual tACCs contain information about the total average number of green- and red-labelled molecules in the OVE, now being the sum of unbound singly labelled molecules and the bound dually labelled complexes. Thus, for the eGFP-labelled MOP receptors, N_g_^total^ = N_g_ + N_gr_, and for the Tomato-labelled 5-HT_1A_ receptors, N_r_^total^ = N_r_ + N_gr_ (Figure 1E). Only the dually labelled receptor-receptor heterodimer molecules give rise to fluorescence intensity fluctuations in both detectors at the same time, and are thus the only ones to contribute to the tCCC, obtained by calculating the cross-correlation function:

(2) $$G_{\mathrm{CC}}\left(\tau \right)=1+\frac{{\langle \delta F}_{\mathrm{green}}\left(t\right)\cdot {\delta F}_{\mathrm{red}}\left(t+\tau \right)\rangle }{\langle F_{\mathrm{green}}\left(t\right)\rangle \langle F_{\mathrm{red}}\left(t\right)\rangle }$$

In contrast to the amplitudes of the tACCs (Equation (1)), which are inversely proportional to the average number of molecules in the OVE (see detailed explanation in [71,75], the zero-lag amplitude of the tCCC (Equation (2)) is directly proportional to the number of dually labelled molecules (N_gr_) and thus increases as N_gr_ increases:

(3) $$G_{\mathrm{CC}}\left(0\right)-1\propto \frac{N_{\mathrm{gr}}}{\left(N_{g}+N_{\mathrm{gr}}\right)\cdot \left(N_{r}+N_{\mathrm{gr}}\right)}\;$$

In order to characterize the degrees of binding between MOP-eGFP and 5-HT_1A_-Tomato, i.e., to determine the number of the heterodimer receptor complexes, FCCS data are further analysed to obtain a dimensionless value known as the relative cross-correlation amplitude (RCCA) [75]. The RCCA is defined as the limiting value, when the lag time approaches zero (τ → 0), of the cross-correlation curve relative to the autocorrelation curve for a single fluorophore. For example, the number of bound, dually labelled molecules carrying both the green and the red label, relative to the total number of molecules carrying the red label (N_r_^total^ = N_r_ + N_gr_), equals the amplitude of the cross-correlation curve (G_CC_(0) − 1) relative to the amplitude of the green autocorrelation curve (G_AC,g_(0) − 1) [75]:

(4) $$\mathrm{RCCA}=\frac{G_{\mathrm{cc}}\left(0\right)-1}{G_{\mathrm{AC},g}\left(0\right)-1}=\frac{N_{\mathrm{gr}}}{N_{r}^{\mathrm{total}}}=\frac{N_{\mathrm{gr}}}{N_{r}+N_{\mathrm{gr}}}$$

Knowing the concentration of MOP-eGFP and 5-HT_1A_-Tomato molecules, and the concentration of heterodimer MOP-eGFP–5-HT_1A_-Tomato complexes, the apparent dissociation constant for the receptor-receptor heterodimer complex can be calculated:

(5) $$K_{d}^{app}=\frac{c_{\mathrm{free}}^{\mathrm{MOP}}\cdot c_{\mathrm{free}}^{5-\mathrm{HT}1A}}{c_{\mathrm{MOP}-5-\mathrm{HT}1A}}$$

or, when expressed using the quantities determined by FCCS:

(6) $$K_{d}^{app}=\frac{\left(N_{g}^{\mathrm{total}}-{\;N}_{r}^{\mathrm{total}}\cdot \mathrm{RCCA}\right)\cdot \left(1-\mathrm{RCCA}\right)}{\mathrm{RCCA}}\cdot \frac{1}{N_{A}\cdot V_{\mathrm{OVE}}}$$

In Equation (6), N_A_ is the Avogadro number. For determining the dynamic range of the RCCA, i.e., the smallest and the largest RCCA values that could be reliably measured, see control experiments described in Supplementary Materials (Section S1. Transfection, positive and negative control cells (Figure S2)). Derivation of Equation (6) is given in Supplementary Materials, Section S2. Calculation of the apparent dissociation constant).

One challenge in dual-color FCCS that is particularly important to consider when fluorescence proteins are being used is the risk of false-positives due to spectral crosstalk between channels, which may lead to overestimation of the cross-correlation amplitude [75]. In order to ascertain that this error is minimized, we have validated the optical setting using control cells—cells expressing eGFP and Tomato were used as negative control (Supplementary Materials, Section S1. Transfection, positive and negative control cells (Figure S2C)) and cells expressing genetically fused eGFP-Tomato were used as positive control (Supplementary Materials, Section S1. Transfection, positive and negative control cells (Figure S2A)). The corresponding tCCCs are shown in Figure 1F. Control experiments showed that the dynamic range in dual-color FCCS differed from the theoretical range, 0 ≤ RCCA ≤ 1, and was determined to be, (0.10 ± 0.07) ≤ RCCA ≤ (0.80 ± 0.08). The RCCA value determined in the negative control experiments, RCCA_nc_ = (0.10 ± 0.07), indicated that only values that are significantly larger then RCCA_nc_ should be considered as a positive indication of binding. Positive control experiments indicated that RCCA values higher than RCCA_pc_ = (0.80 ± 0.08) may not be reached for reasons explained in (Supplementary Materials, Section S1. Transfection, positive and negative control cells (Figure S2E,F), and that RCCA values as high as RCCA_pc_ indicate that 100% binding between the investigated receptors has been reached.

In order to ascertain that errors due to spectral crosstalk are minimized, the optical setting was further validated using the so-called switching mode. In the switching mode, the sample is alternatingly (every 240 µs) illuminated with one laser at a time to excite one fluorophore only [69,71]. By using the switching mode, we could adjust the optical setting so that the crosstalk is minimal when the non-switching mode is being used, as explained in detail in [71], thus ascertaining that increased RCCA are actually being observed following treatment with non-peptide opioids (Supplementary Materials, Section S3. Relative cross-correlation amplitude (RCCA) increased upon opioid treatment. Verification was done by switching FCCS (Figures S3 and S4). Finally, in order to account for the, while minimized, inevitably present cross-talk-induced cross-correlation, the RCCA was corrected by subtracting the cross-talk-induced cross-correlation from the RCCA and scaled up as follows [36,75,76]:

(7) $${\mathrm{RCCA}}_{\mathrm{corrected}}=\frac{\mathrm{RCCA}\;-\;\kappa \cdot f}{1-\;\kappa \cdot f}$$

where κ is the so-called bleed-through ratio, i.e., brightness as reflected by the counts per second and per molecule (CPM) of the green dye in the red channel (CPM_g/r_) when the red fluorophore is not present, divided by its brightness in the green channel CPM_g/g_, κ = CPM_g/r_/CPM_g/g_, and *f* is the count rate (CR) ratio in the green and red channels, *f* = CR_g_/CR_r_. For the optical setting used in our studies, κ = 0.1 and *f* ≤ 1.2. Following treatment, a two-fold increase in eGFP brightness was observed, while Tomato brightness remained largely unchanged (Table 1). To account for this, the κ factor was accordingly scaled. Thus 0.1 ≤ κ ≤ 0.2, and the product 0.12 ≤ κ·*f* ≤ 0.24.

### 4.4. Opioid Treatment

Cells stably expressing MOP-eGFP and 5-HT_1A_-Tomato were cultured in 8-well chambered coverslides (Nalge Nunc International, USA) at 37 °C in a humidified atmosphere containing 5% CO_2_ using phenol red-free media, supplemented in the same way as described above. To ascertain that MOP-eGFP is functional and integrated into cellular physiology, the selective MOP receptor peptide agonist DAMGO ([D-Ala^2^, N-MePhe^4^, Gly-ol^5^] enkephalin), was used (Figure 2) [77]. For experiments with non-peptide opioids, the cells were incubated for 18 h with different concentrations of fentanyl (50 nM, 500 nM, 750 nM or 1 µM) or morphine (250 nM, 500 nM or 750 nM). Based on the results of these experiments (explained in the Section 2), the 750 nM concentration was selected as suitable for further studies. Hence, the cells were subsequently treated with 750 nM codeine or oxycodone. Non-peptide opioids and the peptide opioid DAMGO were all purchased from Sigma-Aldrich.

### 4.5. Intracellular Ca^2+^ Imaging

In order to measure acute opioid-induced changes in Ca^2+^ levels, cells were incubated for 30 min with Fura Red (Invitrogen), a ratiometric Ca^2+^ fluorescent indicator [78,79] stimulated with 750 nM opioids and Ca^2+^ levels were monitored using sequential, i.e., dual-track time-lapse CLSM imaging. In the first track, the 488 nm line of the Ar/ArKr laser was used to excite eGFP and Fura Red. The eGFP signal was collected using the band pass 505-530 nm emission filter, and the Fura Red signal was collected using the long pass 680 nm emission filter. In response to changes in Ca^2+^ concentrations, the excitation wavelength of Fura Red shifts from 472 nm at low Ca^2+^ concentration to 436 nm at high Ca^2+^ concentration [78]. As a result, the intensity of the Fura Red fluorescence signal decreases when intracellular Ca^2+^ levels increase and increases as they fall again. In the second track, the 543 nm HeNe laser was used to excite Tomato, and fluorescence was collected using the band pass 580–620 nm emission filter. The images were collected every 30 s for 40 min (in some cases up to 80 min). The pixel dwell time was 51.2 µs, and the images were collected without averaging.

### 4.6. Western Blotting

Transfected HEK293 cells expressing MOP-eGFP and 5-HT_1A_-Tomato were cultured in collagen-coated T25 flasks (Sarstedt) as described in the *Cell culture and transfection* section. At around 90% confluence, the cells were treated with opioids following the protocol described in the *Opioid treatment* section. Adherent cells were removed from the flasks with trypsin-EDTA (0.05%, Thermo Fisher Scientific), washed with ice-cold PBS and centrifuged at 1500 rpm for 5 min at 4 °C. The cell pellets were solubilized in RIPA lysate buffer (10 × 10^6^ cells/mL) containing protease inhibitor and phosphatase inhibitor (Sigma-Aldrich) and incubated for 30 min on ice. The cell solution was transferred to Eppendorf tubes and centrifuged at 10 000 rpm for 10 min. The total protein content was determined colorimetrically using the BioRad RC DC Protein Assay (Bio-Rad). Samples (20 µg protein) were denatured at 70 °C for 10 min with 4X LDS Sample Buffer, 10X Sample Reducing Agent (Invitrogen) and water was added to a final volume of 30 µL. Samples were loaded on precast polyacrylamide gels (Invitrogen) along with 5 µL of pre-stained standard protein ladder (Thermo Fisher Scientific), electrophoresed and transferred onto a nitrocellulose membrane (Thermo Fisher Scientific). The membrane was probed overnight with antibodies against ERK1/2 (Invitrogen and Cell Signaling Technology, Inc., Danvers, MA, USA), phospho-ERK1/2 (Invitrogen and Cell Signaling Technology, Inc.), p38 (Cell Signaling Technology, Inc.), phospho-p38 (Cell Signaling Technology, Inc.) or β-actin (Invitrogen and Cell Signaling Technology, Inc.). Only one primary antibody was used at a time and the membrane was stripped for 15 min with a western blot stripping buffer (Thermo Fisher Scientific) in between the different primary antibodies. Depending on the primary antibody, either biotin or horseradish peroxidase conjugated secondary antibody (Invitrogen) was used for detection. Western blot experiments were repeated three times, starting from different cell cultures. The same trend was observed in all repetitions. Data from one representative experiment are shown (Supplementary Materials, Section S4. Western blotting (Figure S5)).

### 4.7. LC-MS/MS Opioid Metabolite Analysis

In order to assess whether opioids were active compounds in their own right, HEK293 cells expressing MOP-eGFP and 5-HT_1A_-Tomato were cultured in collagen-coated T25 flasks (Sarstedt) as described in the Section 4.1. At 90% confluence, the cell media was supplemented with 750 nM of morphine, oxycodone, codeine or fentanyl. After 18 h of incubation, the cell media was collected, the cells were lysed, and the cell culture medium and the lysate were stored at −20 °C. Prior to LC-MS/MS analysis, the samples were thawed and allowed to reach room temperature, diluted 10× with MilliQ water and filtered using Agilent/Whatman MiniUniPrep vials 0.2 µm PP (p/n 5190-1421). The samples were aliquoted (in triplicate) and subjected to LC-MS/MS for metabolite analysis. To this aim, a general protocol initially developed for urine analysis was used [80]. An LC-MS/MS analysis tested for the presence of morphine, morphine-3-glucuronide, morphine-6-glucuronide, normorphine, codeine, codeine-6-glucuronide, norcodeine, 6-acetylmorphine and ethylmorphine (Supplementary Materials, Section S5. Liquid chromatography-tandem mass spectrometry (LC-MS/MS) analysis (Tables S1 and S2)).

The analysis was performed using an ACQUITY UPLC I-Class system from Waters (Milford, MA, USA) coupled to a Waters Xevo TQD (Waters). All systems were controlled by MassLynx (Waters, version 4.1 SCN 940). Chromatographic separation was achieved on an ACE Excel 2 C18-PFP column (100 mm × 2.1 mm, 1.8 μm, Aberdeen, Scotland) kept at 60°C. Mobile phase A consisted of 0.001% formic acid in 10 mM ammonium formate pH 5.2 and mobile phase B consisted of 0.001% formic acid in methanol. Initial gradient conditions were 1% B held for 1.5 min, then increasing to 5% B during 0.1 min following a ramping of B to 41% until 7.5 min. The following gradient steps were 95% B until 8 min following 95% B during 1 min before reaching 1% B again and equilibration during 1 min. Total run time was 10.1 min and LC flow was 0.5 mL/min. The injection volume was 3 µL. The electro spray ionization (ESI) interface was operating in positive mode and the mass spectrometer was operating in multiple reaction monitoring (MRM) mode with two transitions for each analyte and one transition for each internal standard according to the table below. Both quadrupoles were set in unit resolution. Data were processed using TargetLynx TM (MassLynx version 4.1 SCN940).

Analytes were identified by their retention time and transition ratio. Quantification of the analytes in the samples was performed using calibration samples with eleven concentrations of the analyte, as shown in Supplementary Materials (Section S6, Table S1).

### 4.8. Statistical Analysis

A student’s *t*-test was used to determine whether the difference between the mean values measured in untreated and treated cells, or in cells treated with different opioids, are significantly different from each other. The results are reported using a two-tailed *p*-value (*p*). The Benjamini–Hochberg method to control the False Discovery Rate (FDR) in sequential modified Bonferroni correction for multiple hypothesis testing was thereafter applied. At an FDR value of 5%, *p* ≤ 0.012 was determined to be statistically significant.

## 5. Conclusions

The long-term usefulness of opioids in chronic pain treatment is hampered by side effects. Drugs targeting 5-HT_1A_ have been shown to alleviate the adverse effects of prolonged opioid use, suggesting interactions between MOP- and 5-HT_1A_-mediated pathways. However, details of underlying mechanisms remain obscure. The aim of our study was to investigate whether these pathways can be integrated at the single-cell level by MOP- and 5-HT_1A_ heterodimerisation. Our quantitative characterization of MOP-eGFP and 5-HT_1A_-Tomato interactions in live cells shows that these receptors can indeed form heterodimers in the plasma membrane of cells expressing physiologically relevant levels of these receptors. Our data show that under the conditions of our study, a surface density of (130 ± 10) molecules/µm^2^, about 33% of the 5-HT_1A_-Tomato receptors are bound in heterodimer complexes with MOP-eGFP in untreated cells.

In line with our hypothesis, we found an increase in MOP–5-HT_1A_ heterodimer formation in cells simultaneously expressing MOP and 5-HT_1A_ receptors following 18 h of incubation with fentanyl, morphine, codeine and oxycodone. All tested non-peptide opioids stabilized the heterodimer complexes and elicited a distinct down-stream cellular signalling response, as evidenced by Ca^2+^ imaging and ERK1/2 and p38 activation. An opioid metabolites analysis did not show any traces of common opioid metabolites, indicating that codeine was an active compound with a similar strength to morphine.

Taken together, our findings suggest that treatments hindering MOP–5-HT_1A_ heterodimer formation could provide potentially new strategies to treat opioid induced hyperalgesia and help to preserve the analgesic effects of opioids. The development of new drugs targeting these mechanisms is therefore of interest.

## Figures and Tables

**Table 1:** Equimolar concentrations of non-peptide opioids differently affect the apparent dissociation constant of MOP–5-HT_1A_, fluorophore brightness, ERK1/2 and p38 activation, and Ca^2+^ levels and signalling dynamics.

Treatment (750 nM)	K_d_ (nM)	CPM_eGFP_ (kHz)	CPM_Tomato_ (kHz)	ERK1/2	p38	CR (kHz)	Ca^2+^ Dynamics
Untreated	440 ± 70	1.1 ± 0.3	0.8 ± 0.2			40	Stationary state
Fentanyl	80 ± 70	1.9 ± 0.7	1.1 ± 0.3	+	+	40	Stationary state
Morphine	200 ± 70	2.0 ± 0.5	1.3 ± 0.7	+	+	120	Small-amplitude oscillations
Codeine	100 ± 70	1.9 ± 0.5	1.0 ± 0.3	+	+	135	Relaxation oscillations
Oxycodone	200 ± 70	1.8 ± 0.7	0.9 ± 0.3	0	+	60	Relaxation oscillations

## Data Availability

Not applicable.

## Supplementary Materials

The following supporting information can be downloaded at: https://www.mdpi.com/article/10.3390/molecules27072350/s1. Subsections: S1. Transfection, positive and negative control cells, S2. Calculation of the apparent dissociation constant (derivation of Equation (6) given in the main text), S3. Relative Cross-Correlation Amplitude (RCCA) increased upon opioid treatment. Verification by switching FCCS, S4. Western blotting and S5. Liquid chromatography-tandem mass spectrometry (LC-MS/MS) analysis. Figures: Figure S1. Transfection and cellular model generation; Figure S2. Determination of the RCCA for positive and negative control cells; Figure S3. RCCA increases upon opioid treatment; Figure S4. Switching FCCS; Figure S5. Western blots showing the activation of extracellular signal-regulated kinase (Erk1/2 and p-Erk1/2) and the p38 mitogen-activated protein kinase (p38/p-p38 MAPK) in HEK293 cells expressing MOP-eGFP and 5-HT_1A_-Tomato. Loading control: β-actin. Tables: Table S1. Calibration standards for LC-MS/MS; Table S2. LC-MS/MS data for morphine (MOR), normorphine (NMOR), Morphine-3-glucuronide (M3G), Morphine-6-glucuronide (M6G), codeine (COD), norcodeine (NCOD), Codeine-6-glucuronide (C6G), Ethyl-morphine (EMOR) and 6-acetyl-morphine (6MAM). For every analyte two transitions (denoted 1 and 2) are measured, where one determines the concentration (underlined) and the other identity. In the measurements internal standards are also measured (denoted D3).

## Sample Availability

Samples of the compounds are not available from the authors.

## References

[1] Collett B. (2011). The burden of chronic pain. Curr. Med. Res. Opin..

[2] Basbaum A.I., Bautista D.M., Scherrer G., Julius D. (2009). Cellular and molecular mechanisms of pain. Cell.

[3] Gosselin R.D., Suter M.R., Ji R.R., Decosterd I. (2010). Glial cells and chronic pain. Neuroscientist.

[4] Kalso E., Edwards J.E., Moore A.R., McQuay H.J. (2004). Opioids in chronic non-cancer pain: Systematic review of efficacy and safety. Pain.

[5] Ballantyne J.C. (2017). Opioids for the Treatment of Chronic Pain: Mistakes Made, Lessons Learned, and Future Directions. Anesth. Analg..

[6] Haleem D.J. (2018). Serotonin-1A receptor dependent modulation of pain and reward for improving therapy of chronic pain. Pharmacol. Res..

[7] Xu X.J., Colpaert F., Wiesenfeld-Hallin Z. (2003). Opioid hyperalgesia and tolerance versus 5-HT1A receptor-mediated inverse tolerance. Trends Pharmacol. Sci..

[8] Colpaert F.C., Deseure K., Stinus L., Adriaensen H. (2006). High-efficacy 5-hydroxytryptamine 1A receptor activation counteracts opioid hyperallodynia and affective conditioning. J. Pharmacol. Exp. Ther..

[9] Rojas-Corrales M.O., Berrocoso E., Micó J.A. (2005). Role of 5-HT1A and 5-HT1B receptors in the antinociceptive effect of tramadol. Eur. J. Pharmacol..

[10] Mico J.A., Berrocoso E., Ortega-Alvaro A., Gibert-Rahola J., Rojas-Corrales M.O. (2006). The role of 5-HT1A receptors in research strategy for extensive pain treatment. Curr. Top. Med. Chem..

[11] Kosek E., Jensen K.B., Lonsdorf T.B., Schalling M., Ingvar M. (2009). Genetic variation in the serotonin transporter gene (5-HTTLPR, rs25531) influences the analgesic response to the short acting opioid Remifentanil in humans. Mol. Pain..

[12] Tour J., Löfgren M., Mannerkorpi K., Gerdle B., Larsson A., Palstam A., Bileviciute-Ljungar I., Bjersing J., Martin I., Ernberg M. (2017). Gene-to-gene interactions regulate endogenous pain modulation in fibromyalgia patients and healthy controls-antagonistic effects between opioid and serotonin-related genes. Pain.

[13] Møller M., Jakobsen S., Gjedde A. (2007). Parametric and regional maps of free serotonin 5HT1A receptor sites in human brain as function of age in healthy humans. Neuropsychopharmacology.

[14] Petrovic P., Ingvar M. (2002). Imaging cognitive modulation of pain processing. Pain.

[15] Martikainen I.K., Hirvonen J., Kajander J., Hagelberg N., Mansikka H., Någren K., Hietala J., Pertovaara A. (2007). Correlation of human cold pressor pain responses with 5-HT(1A) receptor binding in the brain. Brain Res..

[16] Tuominen L., Nummenmaa L., Keltikangas-Järvinen L., Raitakari O., Hietala J. (2014). Mapping neurotransmitter networks with PET: An example on serotonin and opioid systems. Hum. Brain Mapp..

[17] Azmitia E.C., Gannon P.J., Kheck N.M., Whitaker-Azmitia P.M. (1996). Cellular localization of the 5-HT1A receptor in primate brain neurons and glial cells. Neuropsychopharmacology.

[18] Grace P.M., Maier S.F., Watkins L.R. (2015). Opioid-induced central immune signaling: Implications for opioid analgesia. Headache.

[19] Roeckel L.A., Le Coz G.M., Gavériaux-Ruff C., Simonin F. (2016). Opioid-induced hyperalgesia: Cellular and molecular mechanisms. Neuroscience.

[20] Albrecht D.S., Forsberg A., Sandström A., Bergan C., Kadetoff D., Protsenko E., Lampa J., Lee Y.C., Höglund C.O., Catana C. (2019). Brain glial activation in fibromyalgia—A multi-site positron emission tomography investigation. Brain Behav. Immun..

[21] Schrepf A., Harper D.E., Harte S.E., Wang H., Ichesco E., Hampson J.P., Zubieta J.-K., Clauw D.J., Harris R.E. (2016). Endogenous opioidergic dysregulation of pain in fibromyalgia: A PET and fMRI study. Pain.

[22] Kishimoto K., Koyama S., Akaike N. (2001). Synergistic mu-opioid and 5-HT1A presynaptic inhibition of GABA release in rat periaqueductal gray neurons. Neuropharmacology.

[23] Jasmin L., Wu M.V., Ohara P.T. (2004). GABA puts a stop to pain. Curr. Drug Targets CNS Neurol. Disord..

[24] Cussac D., Rauly-Lestienne I., Heusler P., Finana F., Cathala C., Bernois S., De Vries L. (2012). μ-Opioid and 5-HT1A receptors heterodimerize and show signalling crosstalk via G protein and MAP-kinase pathways. Cell Signal..

[25] Liu J.G., Prather P.L. (2001). Chronic exposure to mu-opioid agonists produces constitutive activation of mu-opioid receptors in direct proportion to the efficacy of the agonist used for pretreatment. Mol. Pharmacol..

[26] Tsai R.-Y., Tai Y.-H., Tzeng J.-I., Lin S.-L., Shen C.-H., Yang C.-P., Hsin S.-T., Wang C.-B., Wong C.-S. (2009). Ultra-low dose naloxone restores the antinociceptive effect of morphine in pertussis toxin-treated rats and prevents glutamate transporter downregulation by suppressing the p38 mitogen-activated protein kinase signaling pathway. Neuroscience.

[27] Radoi V. (2019). Interactions between the opioid and serotonin systems in chronic pain. Quantitative Live Cell Study by Fluorescence Cross-Correlation Spectroscopy (FCCS). Ph.D. Thesis.

[28] Hulme E.C., Trevethick M.A. (2010). Ligand binding assays at equilibrium: Validation and interpretation. Br. J. Pharmacol..

[29] Watabe M., Arjunan S.N.V., Chew W.X., Kaizu K., Takahashi K. (2019). Cooperativity transitions driven by higher-order oligomer formations in ligand-induced receptor dimerization. Phys. Rev. E.

[30] Heusler P., Tardif S., Cussac D. (2016). Agonist stimulation at human μ opioid receptors in a [(35)S]GTPγS incorporation assay: Observation of “bell-shaped” concentration-response relationships under conditions of strong receptor G protein coupling. J. Recept. Signal Transduct. Res..

[31] McIntyre I.M., Anderson D.T. (2012). Postmortem Fentanyl Concentrations: A Review. J. Forensic Res..

[32] Goldbeter A. (1996). Biochemical Oscillations and Cellular Rhythms: The Molecular Bases of Periodic and Chaotic Behaviour.

[33] Macey T.A., Lowe J.D., Chavkin C. (2006). Mu opioid receptor activation of ERK1/2 is GRK3 and arrestin dependent in striatal neurons. J. Biol. Chem..

[34] Tan M., Walwyn W.M., Evans C.J., Xie C.W. (2009). p38 MAPK and beta-arrestin 2 mediate functional interactions between endogenous micro-opioid and alpha2A-adrenergic receptors in neurons. J. Biol. Chem..

[35] Kasai R.S., Kusumi A. (2014). Single-molecule imaging revealed dynamic GPCR dimerization. Curr. Opin. Cell Biol..

[36] Petersen J., Wright S., Rodríguez D., Matricon P., Lahav N., Vromen A., Friedler A., Strömqvist J., Wennmalm S., Carlsson J. (2017). Agonist-induced dimer dissociation as a macromolecular step in G protein-coupled receptor signaling. Nat. Commun..

[37] Foster S.R., Bräuner-Osborne H. (2018). Investigating Internalization and Intracellular Trafficking of GPCRs: New Techniques and Real-Time Experimental Approaches. Handb. Exp. Pharmacol..

[38] Kanaya N., Zakhary D.R., Murray P.A., Damron D.S. (1998). Differential effects of fentanyl and morphine on intracellular Ca^2+^ transients and contraction in rat ventricular myocytes. Anesthesiology.

[39] Fan R., Schrott L.M., Snelling S., Ndi J., Arnold T., Korneeva N.L. (2015). Chronic oxycodone induces integrated stress response in rat brain. BMC Neurosci..

[40] Hutchinson M.R., Northcutt A.L., Hiranita T., Wang X., Lewis S.S., Thomas J., Van Steeg K., Kopajtic T.A., Loram L.C., Sfregola C. (2012). Opioid activation of toll-like receptor 4 contributes to drug reinforcement. J. Neurosci..

[41] Al-Hasani R., Bruchas M.R. (2011). Molecular mechanisms of opioid receptor-dependent signaling and behavior. Anesthesiology.

[42] Remillard D., Kaye A.D., McAnally H. (2019). Oxycodone’s Unparalleled Addictive Potential: Is it Time for a Moratorium?. Curr. Pain Headache Rep..

[43] Yue Q.Y., Hasselström J., Svensson J.O., Säwe J. (1991). Pharmacokinetics of codeine and its metabolites in Caucasian healthy volunteers: Comparisons between extensive and poor hydroxylators of debrisoquine. Br. J. Clin. Pharmacol..

[44] Cortazzo M.H., Copenhaver D., Fishman S.M., Benzon H.T., Rathmell J.P., Wu C.L., Turk D.C., Argoff C.E., Hurley R.W. (2013). Major Opioids and Chronic Opioid Therapy. Practical Management of Pain.

[45] Johnson J.L., Rolan P.E., Johnson M.E., Bobrovskaya L., Williams D., Johnson K.F., Tuke J., Hutchinson M. (2014). Codeine-induced hyperalgesia and allodynia: Investigating the role of glial activation. Transl. Psychiatry.

[46] Mignat C., Wille U., Ziegler A. (1995). Affinity profiles of morphine, codeine, dihydrocodeine and their glucuronides at opioid receptor subtypes. Life Sci..

[47] Schmidt H., Vormfelde S.V., Klinder K., Gundert-Remy U., Gleiter C.H., Skopp G., Aderjan R., Fuhr U. (2002). Affinities of dihydrocodeine and its metabolites to opioid receptors. Pharmacol. Toxicol..

[48] Ingelman-Sundberg M. (2005). Genetic polymorphisms of cytochrome P450 2D6 (CYP2D6): Clinical consequences, evolutionary aspects and functional diversity. Pharm. J..

[49] Tzvetkov M.V., dos Santos Pereira J.N., Meineke I., Saadatmand A.R., Stingl J.C., Brockmöller J. (2013). Morphine is a substrate of the organic cation transporter OCT1 and polymorphisms in OCT1 gene affect morphine pharmacokinetics after codeine administration. Biochem. Pharmacol..

[50] Kasai R.S., Suzuki K.G.N., Prossnitz E.R., Koyama-Honda I., Nakada C., Fujiwara T.K., Kusumi A. (2011). Full characterization of GPCR monomer-dimer dynamic equilibrium by single molecule imaging. J. Cell Biol..

[51] Casadó-Anguera V., Moreno E., Mallol J., Ferré S., Canela E.I., Cortés A., Casadó V. (2019). Reinterpreting anomalous competitive binding experiments within G protein-coupled receptor homodimers using a dimer receptor model. Pharmacol. Res..

[52] Jorand R., Biswas S., Wakefield D.L., Tobin S.J., Golfetto O., Hilton K., Ko M., Ramos J.W., Small A.R., Chu P. (2016). Molecular signatures of mu opioid receptor and somatostatin receptor 2 in pancreatic cancer. Mol. Biol. Cell..

[53] Touhara K.K., MacKinnon R. (2018). Molecular basis of signaling specificity between GIRK channels and GPCRs. eLife.

[54] Volpe D.A., Tobin G.A.M., Mellon R.D., Katki A.G., Parker R.J., Colatslcy T., Kropp T.J., Verbois S.L. (2011). Uniform assessment and ranking of opioid Mu receptor binding constants for selected opioid drugs. Regul. Toxicol. Pharmacol..

[55] Rickli A., Liakoni E., Hoener M.C., Liechti M.E. (2018). Opioid-induced inhibition of the human 5-HT and noradrenaline transporters in vitro: Link to clinical reports of serotonin syndrome. Br. J. Pharmacol..

[56] Heiskanen T., Langel K., Gunnar T., Lillsunde P., Kalso E.A. (2015). Opioid Concentrations in Oral Fluid and Plasma in Cancer Patients with Pain. J. Pain Symptom Manag..

[57] Christrup L.L., Foster D., Popper L.D., Troen T., Upton R. (2008). Pharmacokinetics, efficacy, and tolerability of fentanyl following intranasal versus intravenous administration in adults undergoing third-molar extraction: A randomized, double-blind, double-dummy, two-way, crossover study. Clin Ther..

[58] Trescot A.M. (2010). Review of the role of opioids in cancer pain. J. Natl. Compr. Cancer Netw..

[59] Pereira J., Lawlor P., Vigano A., Dorgan M., Bruera E. (2001). Equianalgesic dose ratios for opioids: A critical review and proposals for long-term dosing. J. Pain Symptom Manag..

[60] Rennick A., Atkinson T., Cimino N.M., Strassels S.A., McPherson M.L., Fudin J. (2016). Variability in Opioid Equivalence Calculations. Pain Med..

[61] Hafner M., Niepel M., Chung M., Sorger P.K. (2016). Growth rate inhibition metrics correct for confounders in measuring sensitivity to cancer drugs. Nat. Methods.

[62] Thomas P., Smart T.G. (2005). HEK293 cell line: A vehicle for the expression of recombinant proteins. J. Pharmacol. Toxicol. Methods.

[63] Westerink R.H., Ewing A.G. (2008). The PC12 cell as model for neurosecretion. Acta Physiol..

[64] Khan K.H. (2013). Gene expression in Mammalian cells and its applications. Adv. Pharm. Bull..

[65] Vukojevic V., Heidkamp M., Ming Y., Johansson B., Terenius L., Rigler R. (2008). Quantitative single-molecule imaging by confocal laser scanning microscopy. Proc. Natl. Acad. Sci. USA.

[66] Vukojevic V., Papadopoulos D.K., Terenius L., Gehring W.J., Rigler R. (2010). Quantitative study of synthetic Hox transcription factor-DNA interactions in live cells. Proc. Natl. Acad. Sci. USA.

[67] Schwille P. (2001). Fluorescence correlation spectroscopy and its potential for intracellular applications. Cell Biochem. Biophys..

[68] Vukojević V., Pramanik A., Yakovleva T., Rigler R., Terenius L., Bakalkin G. (2005). Study of molecular events in cells by fluorescence correlation spectroscopy. Cell Mol. Life Sci..

[69] Kim S.A., Heinze K.G., Schwille P. (2007). Fluorescence correlation spectroscopy in living cells. Nat. Methods.

[70] Tian Y., Martinez M.M., Pappas D. (2011). Fluorescence correlation spectroscopy: A review of biochemical and microfluidic applications. Appl. Spectrosc..

[71] Rogacki M.K., Golfetto O., Tobin S.J., Li T., Biswas S., Jorand R., Zhang H., Radoi V., Ming Y., Svenningsson P. (2018). Dynamic lateral organization of opioid receptors (kappa, mu_wt_ and mu_N40D_) in the plasma membrane at the nanoscale level. Traffic.

[72] Schwille P., Ries J. (2011). Principles and Applications of Fluorescence Correlation Spectroscopy (Fcs). Biophotonics: Spectroscopy, Imaging, Sensing, and Manipulation.

[73] Bacia K., Kim S.A., Schwille P. (2006). Fluorescence cross-correlation spectroscopy in living cells. Nat. Methods.

[74] Krieger J.W., Singh A.P., Bag N., Garbe C.S., Saunders T., Langowski J., Wohland T. (2015). Imaging fluorescence (cross-) correlation spectroscopy in live cells and organisms. Nat. Protoc..

[75] Bacia K., Petrášek Z., Schwille P. (2012). Correcting for spectral cross-talk in dual-color fluorescence cross-correlation spectroscopy. Chemphyschem.

[76] Du Z., Yu J., Li F., Deng L., Wu F., Huang X., Bergstrand J., Widengren J., Dong C., Ren J. (2018). In Situ Monitoring of p53 Protein and MDM2 Protein Interaction in Single Living Cells Using Single-Molecule Fluorescence Spectroscopy. Anal. Chem..

[77] Xu H., Partilla J.S., Wang X., Rutherford J.M., Tidgewell K., Prisinzano T.E., Bohn L.M., Rothman R.B. (2007). A comparison of noninternalizing (herkinorin) and internalizing (DAMGO) mu-opioid agonists on cellular markers related to opioid tolerance and dependence. Synapse.

[78] Burchiel S.W., Edwards B.S., Kuckuck F.W., Lauer F.T., Prossnitz E., Ransom J.T., Sklar L.A. (2000). Analysis of free intracellular calcium by flow cytometry: Multiparameter and pharmacologic applications. Methods.

[79] Tinning P.W., Franssen A.J.P.M., Hridi S.U., Bushell T.J., McConnell G. (2018). A 340/380 nm light-emitting diode illuminator for Fura-2 AM ratiometric Ca^2+^ imaging of live cells with better than 5 nM precision. J. Microsc..

[80] Murphy C.M., Huestis M.A. (2005). LC-ESI-MS/MS analysis for the quantification of morphine, codeine, morphine-3-beta-D-glucuronide, morphine-6-beta-D-glucuronide, and codeine-6-beta-D-glucuronide in human urine. J. Mass Spectrom..

