# Supplementary material for: Non-Peptide Opioids Differ in Effects on Mu-Opioid (MOP) and Serotonin 1A (5-HT1A) Receptors Heterodimerization and Cellular Effectors (Ca2+, ERK1/2 and p38) Activation
Source: Molecules. 2022 Apr 6;27(7):2350. doi: 10.3390/molecules27072350 (PMC9000251; doi:10.3390/molecules27072350)
Supplement: Supplementary file 1 [file molecules-27-02350-s001.zip › molecules-1608654-supplementary.pdf]

molecules

MDPI

Supplementary Information

Non-peptide opioids differ in effects on mu-opioid (MOP) and serotonin 1A (5-HT1A) receptors heterodimerization and cellular effectors (Ca^{2+}, ERK1/2 and p38) activation

Vlad Radoi [1], Gerd Jakobsson [2], Vinko Palada [3], Andrej Nikosjkov [1], Henrik Druid [2,4], Lars Terenius [1], Eva Kosek [1,5] and Vladana Vukojević [1,*]

[1] Department of Clinical Neuroscience, Karolinska Institute, 171 76 Stockholm, Sweden
[2] Department of Forensic Genetics and Forensic Toxicology, National Board of Forensic Medicine, 587 58, Linköping, Sweden
[3] Department of Physiology and SleepWell Research Program, Faculty of Medicine, University of Helsinki, 00290 Helsinki, Finland
[4] Department of Oncology-Pathology, Karolinska Institute, 171 77 Stockholm, Sweden
[5] Department of Surgical Sciences, Uppsala University, 752 36 Uppsala, Sweden
* Correspondence: vladana.vukojevic@ki.se

Citation: Radoi, V.; Jakobsson, G.; Palada, V.; Nikosjkov, A.; Druid, H.; Terenius, L.; Kosek, E.; Vukojević, V. Non-peptide Opioids Differ in Effects on Mu-Opioid (MOP) and Serotonin 1A (5-HT1A) Receptors Heterodimerization and Cellular Effectors (Ca^{2+}, ERK1/2 and p38) Activation. *Molecules* **2022**, *27*, 2350. https://doi.org/10.3390/molecules27072350

Academic Editors: Mariana Spetea and Richard M. van Rijn

Received: 7 February 2022
Accepted: 2 April 2022
Published: 8 April 2022

Publisher's Note: MDPI stays neutral with regard to jurisdictional claims in published maps and institutional affiliations.

Copyright: © 2022 by the authors. Licensee MDPI, Basel, Switzerland. This article is an open access article distributed under the terms and conditions of the Creative Commons Attribution (CC BY) license (https://creativecommons.org/licenses/by/4.0/).

Abstract: Importance of the dynamic interplay between the opioid and the serotonin neuromodulatory systems in chronic pain is well recognized. In this study, we investigated whether these two signaling pathways can be integrated at the single-cell level *via* direct interactions between the mu-opioid (MOP) and the serotonin 1A (5-HT1A) receptors. Using Fluorescence Cross-Correlation Spectroscopy (FCCS), a quantitative method with single-molecule sensitivity, we characterized in live cells MOP and 5-HT1A interactions and the effects of prolonged (18 h) exposure to selected non-peptide opioids: morphine, codeine, oxycodone and fentanyl, on the extent of these interactions. The results indicated that MOP and 5-HT1A receptors form in the plasma membrane heterodimers that are characterized with an apparent dissociation constant K_d^{app} = (440 ± 70) nM). Prolonged exposure to all non-peptide opioids tested facilitated MOP and 5-HT1A heterodimerization and stabilized the heterodimer complexes, albeit to a different extent: $K_{d,Fentanyl}^{app}$ = (80 ± 70) nM), $K_{d,Morphine}^{app}$ = (200 ± 70) nM, $K_{d,Codeine}^{app}$ = (100 ± 70) nM and $K_{d,Oxycodone}^{app}$ = (200 ± 70) nM. The non-peptide opioids differed also in the extent to which they affected the mitogen-activated protein kinases (MAPKs) p38 and the extracellular signal-regulated kinase (Erk1/2), with morphine, codeine and fentanyl activating both pathways, whereas oxycodone activated p38 but not ERK1/2. Acute stimulation with different non-peptide opioids differently affected the intracellular Ca^{2+} levels and signalling dynamics. Hypothetically, targeting MOP–5-HT1A heterodimer formation could become a new strategy to counteract opioid induced hyperalgesia and help to preserve the analgesic effects of opioids in chronic pain.

Keywords: Chronic pain, Fluorescence Cross-Correlation Spectroscopy (FCCS), G protein-coupled receptor (GPCR), Opioid, Serotonin

S1. Transfection, positive and negative control cells

To generate HEK293 and PC12 cell lines that stably produce the mu-opioid receptor fused at the C-terminus with the enhanced Green Fluorescent Protein (MOP-eGFP) and the serotonin 1A receptor fused at the C-terminus with the Red Fluorescent Protein variant Tomato (5-HT1A-Tomato), the pBudCE4.1 vector (Fig. S1A) is used to enable independent expression of two genes from a single plasmid and ensure that there is an equivalent copy number of each gene in the cell.

CLSM images of stably transformed HEK293 cells (main text, Fig. 1B) and PC12 cells (Fig. S1 B), showed that both, MOP-eGFP and 5-HT_{1A}-Tomato receptors, were expressed, successfully trafficked, and inserted in the plasma membrane (main text Fig. 1B, Fig. 2, Fig. S1A). While Tomato accumulation in granular cytoplasmic structures, presumably trafficking vesicles shuttling the 5-HT_{1A}-Tomato receptor to/from the plasma membrane, was observed in PC12 cells (Fig. 2A and B, and Fig. S1 B), this was not observed to the same extent in HEK293 cells (Fig. 1B). Receptor functionality was validated by examining the effects of treatment with selected agonists of MOP and 5-HT_{1A} receptors and their combination on the phosphorylation of mitogen-activated protein kinases (MAPKs) p38 and extracellular signal-regulated kinase (Erk1/2). The data show that all tested compounds and their combination increase the protein levels of ERK1/2, p-ERK1/2, p38 and p-p38 as compared to their levels in untreated cells (Fig. S1 C).

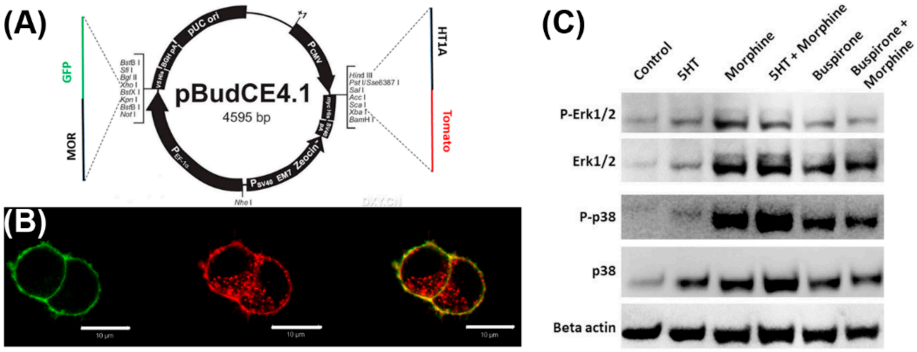

Figure S1. Transfection and cellular model generation. A. Cloning vector for the expression of genes encoding for the mu-opioid receptor fused at the C-terminus with the enhanced Green Fluorescent Protein (MOP-eGFP) and the serotonin 1A receptor fused at the C-terminus with the red fluorescent protein Tomato (5-HT_{1A}-Tomato). **B.** CLSM image of PC12 cells genetically modified to stably express MOP-eGFP (green) and 5-HT_{1A}-Tomato (red). Scale bar 10 μm. **C.** Western blot analysis of phosphorylation of MAPKs ERK1/2, p-ERK1/2, p38 and P-p38 in cells expressing MOP-eGFP and 5-HT_{1A}-Tomato, following 18 h treatment with: serotonin (5HT), morphine or buspirone and serotonin or buspirone in combination with morphine. β-actin is used as a loading control.

To generate positive control cells, eGFP sequence from the pEGFPN1 vector was cut using 5′KpnI and 3′AgeI restriction enzymes, cloned into the ptd-Tomato-N1 vector and conjoined to the N-terminus of tdTomato via the HindIII linker. The thus modified ptd-Tomato-N1 vector was transfected into HEK293 and PC12 cells using the Lipofectamine 2000 transfection reagent. Cells stably expressing the linked eGFP-dTomato construct were isolated through selection with Geneticine antibiotic (250 μg /mL), imaged (Fig. S2A, exemplifying a HEK293 cell) and subjected to FCCS (Fig. S2 B). Cells expressing free eGFP and dTomato were used as a negative control (Fig. S2 C, exemplifying a PC12 cell). To express free eGFP, the pEGFPN1 vector was used. To express free dTomato, the ptd-Tomato-N1 vector was modified to allow free dTomato expression. To generate the negative control cells, transfection was performed using equal amounts of these vectors at the same time.

While positive and negative control cells could not be distinguished by CLSM imaging (Fig. S2 A and C, respectively), FCCS showed a clear distinction with respect to the cross-correlation amplitude (main text Fig. 1F and Fig. S2 B versus D) and with respect to the relative cross-correlation amplitude (RCCA, Fig. S2 E and F). To determine the RCCA, the ratio between the cross-correlation amplitude ($G_{CC}(\tau)$-1) and the autocorrelation amplitude in the green channel ($G_{AC,g}(\tau)$-1) was calculated for all lag times and plotted as a function of the lag time (Fig. S2E), and an average value for time points between 10 – 100 μs (1×10^{-5} s – 1×10^{-4} s) was calculated as representative for the RCCA at zero lag time (Fig. S2E).

Theoretically, $0 \leq RCCA \leq 1$, but the determined RCCAs values for the negative and the positive control cells define, respectively, the practical minimum, $RCCA_{nc} = 0.10 \pm 0.07$,

indicating no binding, and the practical maximum, $RCCA_{cc}$ = 0.80 ± 0.08, indicating 100 % binding (Fig. S2F). Positive and negative control cells were generated using both, HEK293 and PC12 cells. Difference between corresponding RCCA values measured in HEK293 and PC12 cells was not observed, *i.e.*, $RCCA_{nc}^{HEK293} \approx RCCA_{nc}^{PC12}$ and $RCCA_{pc}^{HEK293} \approx RCCA_{pc}^{PC12}$.

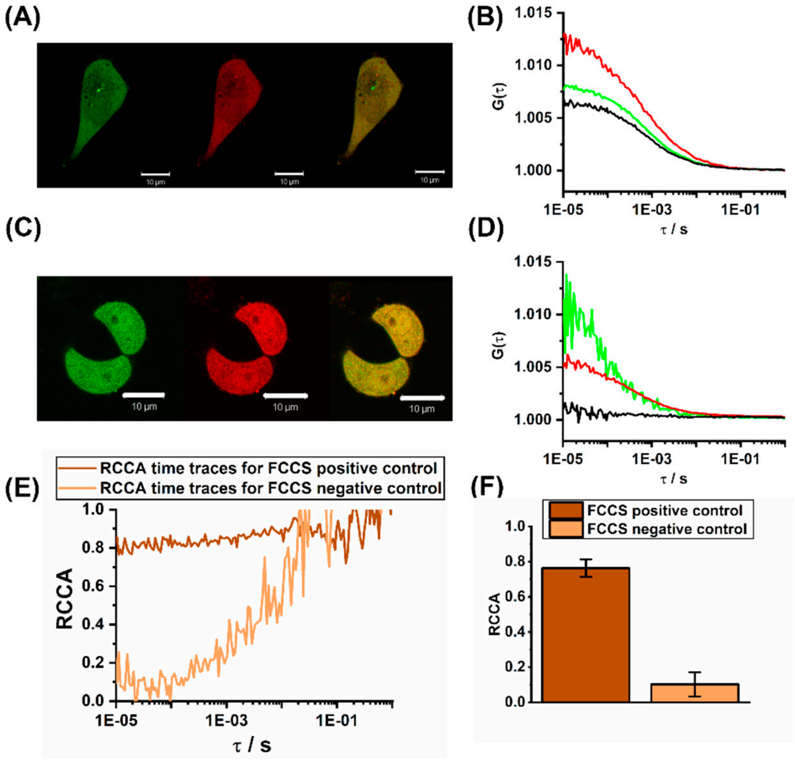

Figure S2. Determination of the RCCA for positive and negative control cells. A. CLSM image of a positive control HEK293 cell expressing the linked eGPF-Tomato construct. **B.** FCCS measurement performed on a positive control cell. **C.** CLSM image of a negative control PC12 cells expressing free eGFP (green) and Tomato (red). **D)** FCCS measurement performed on a negative control cell. **E)** RCCA values for positive (black) and negative (grey) control cells as a function of lag time. The RCCA was determined as the limiting value, when $\tau \rightarrow 0$, of the ratio of the cross-correlation amplitude ($G_{CC}(\tau)$-1) and the autocorrelation amplitude in the green channel ($G_{AC,g}(\tau)$-1) for the corresponding lag time (Eq. 3 in the main text). The RCCA for the positive control cells ($RCCA_{pc}$) corresponds to 100 % binding. Conversely, the zero lag time RCCA for the negative control cells ($RCCA_{nc}$), reflects independent eGFP and Tomato diffusion through the observation volume, i.e. zero binding. **F)** RCCA at lag time zero, determined as an average of RCCAs from the time interval 10 μs – 100 μs. The zero lag time RCCA for positive control cells, $RCCA_{pc}$ = (0.80 ± 0.08), and the negative control cells, $RCCA_{nc}$ = (0.10 ± 0.07).

The fact that $RCCA_{pc} < 1$ indicates that in a fraction of eGFP-Tomato molecules only one of the fluorophores is fluorescent. This is not uncommon, and several processes may lead to fluorophores residing in dark states. The fact that $RCCA_{nc} > 0$, indicates that cross-talk, while minimized, is not entirely abolished. However, the RCCA were corrected to account for the spectral crosstalk, as described in eq. (7) and only RCCA values that are significantly larger than $RCCA_{nc}$ were considered for further analysis.

S2. Calculation of the apparent dissociation constant (derivation of equation (6) given in the main text)

The apparent dissociation constant for MOP-eGFP and 5-HT1A-Tomato binding, eq. (6), was derived as follows:

$$K_d = \frac{[MOP-eGFP]_{free} \cdot [5-HT1A-Tomato]_{free}}{[MOP-eGFP-5-HT1A]}, \quad (6a)$$

where $[MOP\text{-}eGFH]_{free}$ and $[5\text{-}HT_{1A}\text{-}Tomato]_{free}$ are concentrations of free receptors, MOP-eGFP and $5\text{-}HT_{1A}$-Tomato, respectively, and $[MOP\text{-}eGFP–5\text{-}HT_{1A}\text{-}Tomato]$ is the concentration of receptor-receptor complexes.

For both receptors, MOP-eGFP and $5\text{-}HT_{1A}$-Tomato, the concentration of free receptor molecules can be expressed as the difference between the total concentration and the concentration of receptor-receptor complexes, rendering equation (6a) into:

$$K_d = \frac{([MOP-eGFP]_{total} - [MOP-eGFP-5-HT1A]) \cdot ([5-HT1A-Tomato]_{total} - [MOP-eGFP-5-HT1A])}{[MOP-eGFP-5-HT1A]} \quad (6b)$$

If we divide both, the numerator and the denumerator in eq. (6b) by the total concentration of red-labeled molecules:

$$K_d = \frac{\frac{([MOP-eGFP]_{total} - [MOP-eGFP-5-HT1A]) \cdot ([5-HT1A-Tomato]_{total} - [MOP-eGFP-5-HT1A])}{[5-HT1A-Tomato]_{total}}}{\frac{[MOP-eGFP-5-HT1A]}{[5-HT1A-Tomato]_{total}}}, \quad (6c)$$

and rearrange eq. (6c), the following expression is obtained:

$$K_d = \frac{([MOP-eGFP]_{total} - [MOP-eGFP-5-HT1A]) \cdot \left(1 - \frac{[MOP-eGFP-5-HT1A]}{[5-HT1A-Tomato]_{total}}\right)}{\frac{[MOP-eGFP-5-HT1A]}{[5-HT1A-Tomato]_{total}}} \quad (6d)$$

By expressing the molar concentration in terms of the average number of molecules in the OVE, $c = n/V_{OVE}$, where n is the number of moles, $n = (N/N_A)$, eq. (6d) becomes:

$$K_d = \frac{\left(\frac{N_g^{total}}{N_A \cdot V_{OVE}} - \frac{N_{gr}}{N_A \cdot V_{OVE}}\right) \cdot \left(1 - \frac{N_{gr}}{N_r^{total}}\right)}{\frac{N_{gr}}{N_r^{total}}}, \quad (6e)$$

which after introducing eq. (4), $RCCA = \frac{N_{rg}}{N_r^{total}}$, gives eq. (6):

$$K_d = \frac{(N_g^{total} - N_{gr}) \cdot \frac{1}{N_A \cdot V_{OVE}} \cdot (1 - RCCA)}{RCCA} = \frac{(N_g^{total} - N_r^{total} \cdot RCCA) \cdot (1 - RCCA)}{RCCA} \cdot \frac{1}{N_A \cdot V_{OVE}} \quad (6)$$

S3. Relative Cross-Correlation Amplitude (RCCA) increased upon opioid treatment. Verification by switching FCCS

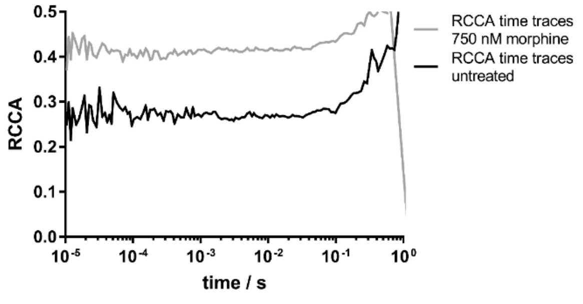

Figure S3. RCCA increases upon opioid treatment. The RCCA in untreated HEK293 cells expressing MOP-eGFP and 5-HT1A-Tomato (black) and after 18 h treatment with 750 nM morphine (grey).

In order to validate the results obtained by FCCS and ascertain that the obtained results are not artifacts due to spectral cross-talk from the green to the red channel, confirmatory experiments using alternating excitation, i.e., switching FCCS (Rogacki et al., 2018), were carried out in both, HEK293 and PC12 cells (Fig. S4).

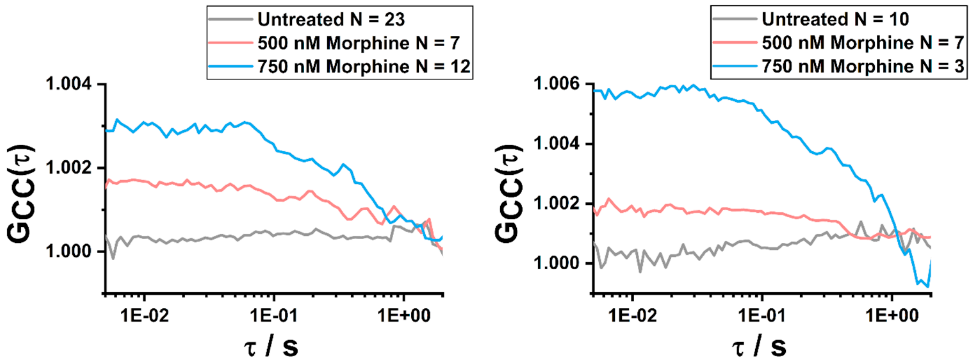

Figure S4. Switching FCCS. Cross-correlation Curves (CCCs) obtained using alternating excitation show in HEK293 (left) and in PC12 (right) cells expressing MOP-eGFP and 5-HT1A-Tomato, that the amplitude of the CCC increases when the cells are treated with increasing concentrations of opioids, here morphine. The switching time was 240 µs.

S4. Western blotting

Western blotting experiments were performed in HEK293 cells (Fig. S5). The measurements were repeated three times. Similar trends were observed in all repetitions.

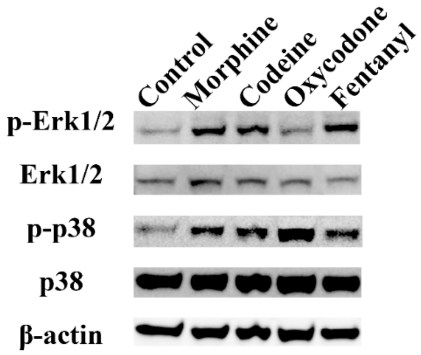

Figure S5. Western blots showing the activation of extracellular signal-regulated kinase (Erk1/2 and p-Erk1/2) and the p38 mitogen-activated protein kinase (p38/p-p38 MAPK) in HEK293 cells expressing MOP-eGFP and 5-HT1A-Tomato. Loading control: β-actin.

S5. Liquid chromatography-tandem mass spectrometry (LC-MS/MS) analysis

Presence of analytes in the samples was confirmed by retention time and transition ratio. The amount was determined using calibration standards of 11 different concentrations prepared in human urine (Table S1).

Table S1. Calibration standards for LC-MS/MS.

Analyte	Calibration range (µg/ml)	Limit of quantification
Morphine	0.025-50	0.025
Normorphine	0.025-5	0.05
Morphine-3-glucuronide	0.1-100	0.2
Morphine-6-glucuronide	0.1-100	0.2
Codeine	0.025-25	0.025
Norcodeine	0.025-5	0.05
Codeine-6-glucuronide	0.1-100	0.2
6-acetyl-morphine	0.01-10	0.01
Ethyl-morphine	0.01-25	0.02

In the blank sample, no analyte was detected. In cell lysate, no analyte was detected. In cell medium, morphine and codeine were detected in correspondingly treated samples (collected data shown in Table S2).

Table S2. LC-MS/MS data for morphine (MOR), normorphine (NMOR), Morphine-3-glucuronide (M3G), Morphine-6-glucuronide (M6G), codeine (COD), norcodeine (NCOD), Codeine-6-glucuronide (C6G), Ethyl-morphine (EMOR) and 6-acetyl-morphine (6MAM). For every analyte two transitions (denoted 1 and 2) are measured, where one determines the concentration (underlined) and the other identity. In the measurements internal standards are also measured (denoted D3).

Sequence 1. 8 mass of pairs

Compound name	Parent (m/z)	Daughter (m/z)	Dwell (s)	Cone (V)	Collision (V)
NMOR 1	272.05	<u>165.00</u>	0.038	50	40
NMOR 2	272.05	121.00	0.038	50	28
M3G 1	462.11	<u>286.15</u>	0.038	50	34
M3G 2	462.11	165.10	0.038	50	68
M6G 1	462.10	<u>286.15</u>	0.038	60	36
M6G 2	462.10	165.10	0.038	60	64
M6G D3	465.11	289.15	0.038	60	34
M3G D3	465.10	289.15	0.038	50	32

Sequence 2. 9 mass of pairs

Compound name	Parent (m/z)	Daughter (m/z)	Dwell (s)	Cone (V)	Collision (V)
NCOD 1	286.10	<u>165.05</u>	0.034	46	44
NCOD 2	286.10	121.00	0.034	46	28
MOR 1	286.05	<u>165.00</u>	0.034	54	36
MOR 2	286.05	201.05	0.034	54	26
C6G 1	476.15	<u>300.10</u>	0.034	62	34
C6G 2	476.15	165.00	0.034	62	64
NCOD D3	289.11	165.05	0.034	50	40
MOR D3	289.10	165.05	0.034	50	42
C6G D3	479.15	303.15	0.034	60	34

Sequence 3. 8 mass of pairs

Compound name	Parent (m/z)	Daughter (m/z)	Dwell (s)	Cone (V)	Collision (V)
COD 1	300.10	<u>165.00</u>	0.038	56	40
COD 2	300.10	215.05	0.038	56	26
EMOR 1	314.10	<u>165.05</u>	0.038	52	46
EMOR 2	314.10	229.10	0.038	52	26
6MAM 1	328.10	<u>165.05</u>	0.038	50	42
6MAM 2	328.10	211.05	0.038	50	28
COD D3	303.15	165.05	0.038	50	44
6MAM D3	331.10	165.05	0.038	50	40
